# Joint species‐trait distribution modeling: The role of intraspecific trait variation in community assembly

**DOI:** 10.1002/ecy.70174

**Published:** 2025-09-04

**Authors:** Nerea Abrego, Pekka Niittynen, Julia Kemppinen, Otso Ovaskainen

**Affiliations:** ^1^ Department of Biological and Environmental Science University of Jyväskylä Jyväskylä Finland; ^2^ Botany and Mycology Unit, Finnish Museum of Natural History University of Helsinki Helsinki Finland; ^3^ Organismal and Evolutionary Biology Research Programme, Faculty of Biological and Environmental Sciences University of Helsinki Helsinki Finland

**Keywords:** Alpine community, community assembly, community modeling, *Empetrum*, leaf area, phenotypic variation, plant height, specific leaf area, trait hierarchy

## Abstract

The links between intraspecific trait variation and community assembly remain little studied, partially due to the lack of statistical methods to jointly model intraspecific trait variation and species abundances at the community level. Here, we extend the joint species distribution modeling (JSDM) framework into the joint species‐trait distribution modeling (JSTDM) framework to explicitly link species abundances to phenotypic variation in traits for multiple species simultaneously. Using a case study of 65 tundra plant species abundances and 3 key functional traits measured across 325 sites, we show how the JSTDM approach (1) estimates the statistical associations among species abundances, species‐level traits, and site‐level traits, relative to environmental variation; (2) improves predictions on trait variation by using information on species abundances; and (3) generates hypotheses about trait‐driven community assembly mechanisms. The JSTDM methodology presented in this study allows assessing the interplay between species abundances and traits at the community level, providing the much needed modeling tools to quantify the role of phenotypic trait variation in eco‐evolutionary community assembly.

## INTRODUCTION

Intraspecific phenotypic trait variation can result from heritable genetic differences among individuals or phenotypic plasticity in relation to environmental conditions. Intraspecific trait variation has been on the main agenda of evolutionary biology for centuries since it provides a basic starting point for studying natural selection. By contrast, how intraspecific trait variation influences community dynamics has only a recent history in ecological research (Bolnick et al., 2011; Laughlin et al., 2012). Theory suggests that intraspecific trait variation can influence community dynamics by influencing the fitness of individuals under varying abiotic conditions and by changing the competitive and other interactive outcomes among individuals (Bolnick et al., 2011). Integrating intraspecific trait variation in community‐level studies is thus a key component to deepen the understanding of community assembly (Bolnick et al., 2011; Violle et al., 2012). An increasing body of empirical research indicates that intraspecific trait variation can indeed play a major role in driving the assembly of ecological communities (Bennett et al., 2016; Kuppler et al., 2020; Siefert et al., 2015; Wong & Carmona, 2021), yet the links between trait variation and community assembly remain insufficiently understood (Gonçalves‐Souza et al., 2023).

The role of intraspecific trait variation in community assembly has been particularly studied for plant communities (Siefert et al., 2015; Violle et al., 2012). Plant traits can respond to abiotic conditions, such as light and resource availability (Freschet et al., 2015; Poorter et al., 2009), as well as to the presence of neighboring individuals (Carmona, Bello, et al., 2019). When soil nutrients are limited, intraspecific trait variation may be reduced due to directional selection, by, for example, reducing specific leaf area which allows for a more efficient or conservative use of resources (Wright et al., 2004). However, competition for the limiting resources will exert further pressure on the availability of those resources, and thus increased intraspecific variation due to the changing abiotic environment can then reciprocally arise as a response to the competitive environment (Bennett et al., 2016; Doudová & Douda, 2020).

As a response to the theoretical and empirical calls for incorporating intraspecific trait variation into community‐level studies, there has been an increasing interest in developing methodological approaches to assess intraspecific trait variation at the community level (Carmona, De Bello, et al., 2019; Cianciaruso et al., 2009; De Bello et al., 2011; Fontana et al., 2016; Valladares et al., 2006; Wong & Carmona, 2021). On the one hand, several authors have suggested indices that quantify the amount and nature of intraspecific trait variation among individuals (Cianciaruso et al., 2009; Fontana et al., 2016) or among populations and species (Carmona, De Bello, et al., 2019; Valladares et al., 2006). Such indices can be linked to environmental variation, species richness, and community composition (De Bello et al., 2011; Wong & Carmona, 2021). On the other hand, intraspecific trait variation can be assessed directly by models that predict trait values across environmental gradients (Sandel et al., 2021), or conversely, that predict species abundances from the spatially varying trait values (Laughlin et al., 2012). In this line, Clark (2016) introduced two multivariate approaches called the trait response model (TRM) and the predictive trait model (PTM). TRM computes community‐weighted mean traits per site as the multivariate response, while PTM predicts species abundances via a joint species distribution model and uses species‐level traits to predict trait distributions. However, TRM and PTM rely solely on species‐level trait data and site‐level abundances, without accounting for intraspecific trait variation. Hence, what has been lacking are models that would jointly predict both trait values and species abundances along environmental gradients and thus statistically link species‐level and site‐level trait values to abundance variation at the community level. Such models would be much needed to better understand the complexity of the mechanisms through which intraspecific trait variation shapes ecological communities.

In this study, we developed a new statistical framework to jointly model variation in site‐level traits and species abundances for species‐rich communities. We present our approach as an extension to joint species distribution modeling (JSDM). JSDM has recently emerged in the ecological literature as a tool to model how species abundances vary over environmental and spatial gradients and to detect statistical associations in co‐occurrence or co‐abundance while controlling for the direct effect of environmental variation (Ovaskainen & Abrego, 2020; Warton et al., 2015). While existing JSDM approaches have been used to ask how species traits and phylogenetic relationships influence the environmental responses, thus far JSDM has incorporated data on species‐level traits without accounting for intraspecific trait variation (Abrego et al., 2017; Brown et al., 2014; Pollock et al., 2012). Here, we extend the JSDM methodology into joint species‐trait distribution modeling (JSTDM) to ask how variation in intraspecific trait values relates not only to environmental variation but also to the abundance and species‐level trait variation.

To illustrate our approach, we use data on tundra plant communities of 65 species sampled over elevational gradients. Elevational gradients offer classical study systems to investigate how communities respond to sharp changes in nutrient and climatic conditions, which become harsher for plant growth at higher elevations (Körner, 2007). Not only do plant species composition and richness change markedly along elevational gradients (Rahbek, 1995; Sundqvist et al., 2013) but also intraspecific trait distributions (Rixen et al., 2022). We defined a JSTDM that simultaneously models variation in species abundances and three key functional traits of tundra plants: median height, specific leaf area (the ratio of leaf area to dry mass), and leaf area (the total surface area of a leaf), which are known to be influenced by the microclimatic variations across sub‐arctic landscapes (Kemppinen & Niittynen, 2022).

Using a latent variable approach, the JSTDM approach estimates statistical associations among species abundances and species‐ and site‐level traits to capture signatures of trait‐driven intra‐ and inter‐specific interactions. Competitive trait hierarchies may, for example, lead to negative associations between a given trait value of the neighboring species and the abundances of a given species (Kraft et al., 2014). We used the model to address two key questions about the link between trait variation and abundance variation. First, motivated by the fact that site‐level trait data are much more resource‐intensive to acquire in the field than data on species abundance, we asked to what extent community‐level knowledge on species abundances helps in predicting species traits. To address this question, we split the sites into training and test, fitted the JSTDM model to data on training sites, and then predicted traits for the test sites, assuming that information on species abundances on those test sites was either available or not available. Second, to evaluate the link between trait variation and community assembly, we asked the other way around whether traits influenced the abundances and richness of the neighboring species. For these predictions, we selected *Empetrum nigrum* as the focal species, as it is well known to affect the performance and composition of the neighboring plant communities (Bråthen et al., 2010; le Roux et al., 2013; Mod et al., 2016; Pellissier et al., 2010). We conducted scenario simulations where we varied both the environmental conditions and the presence, abundance, and trait values of *E. nigrum* to quantify the relative roles of each of these in the assembly of the local community.

## METHODS

### The JSTDM framework

We implemented the JSTDM framework as an extension of the Hierarchical Modeling of Species Communities (HMSC, Ovaskainen & Abrego, 2020; Ovaskainen, Tikhonov, Norberg, et al., 2017). HMSC is a flexible joint species distribution model (JSDM) that enables integration of data on species abundances, environmental covariates, and species‐level traits and phylogenies while explicitly accounting for the spatiotemporal context of the study. HMSC is a hierarchical multivariate generalized linear mixed model, where the element $Y_{\mathrm{ij}}$ of the response matrix Y contains the abundance of the species j for the site i. The linear predictor is modeled as $L_{\mathrm{ij}}=\sum_{k}^{n_{c}}x_{\mathrm{ik}}\beta_{\mathrm{kj}}+\sum_{k}^{n_{f}}\eta_{\mathrm{ik}}\lambda_{\mathrm{kj}}$, where the $\beta_{\mathrm{kj}}$ are the responses of the species to the $k=1,\ldots ,n_{c}$ measured predictors $x_{\mathrm{ik}}$, and the $\lambda_{\mathrm{kj}}$ are the latent loadings, that is, the responses of the species to the $k=1,\ldots ,n_{f}$ latent factors $\eta_{\mathrm{ik}}$. The expected response $\mu_{\mathrm{kj}}=E\left[\beta_{\mathrm{kj}}\right]$ of species j to environmental predictor k is modeled through species‐level traits, $\mu_{\mathrm{kj}}=\sum_{l}^{n_{t}}t_{\mathrm{jl}}\gamma_{\mathrm{kl}},$ where $t_{\mathrm{jl}}$ is the trait l of species j, and $\gamma_{\mathrm{kl}}$ is the effect of trait l to predictor k (Abrego et al., 2017). The latent loadings can be translated into the species‐to‐species covariance matrix Ω, the element $\Omega_{j_{1},j_{2}}=\sum_{k}^{n_{f}}\lambda_{kj_{1}}\lambda_{kj_{2}}$ describing to what extent the species $j_{1}$ and $j_{2}$ covary in their abundance, beyond to the level of covariation induced by their responses to the measured predictors (for more information about HMSC, see Appendix S1: Section S1).

**FIGURE 1:**
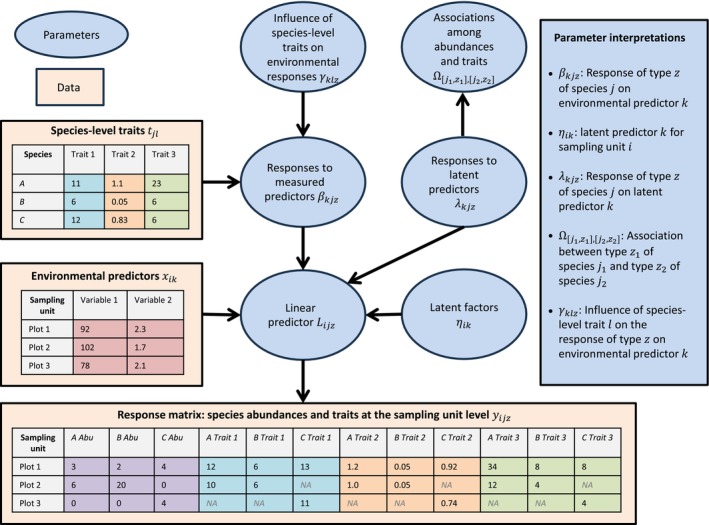
Input data and model parameters estimated in joint species‐trait distribution modeling (JSTDM). The data consist of three input matrices: (1) the multiresponse matrix **Y** including for each species the site‐level abundances and traits values; (2) the matrix **X** of the site‐level environmental predictors; and (3) the trait matrix **T** including the species‐level traits (e.g., averages across all sites) for each species. Note that when a species is not recorded in a given site and thus trait values cannot be measured, these are indicated as missing values (NA) in the multiresponse matrix. As outputs, JSTDM estimates the parameters summarized in the figure and mathematically described in *Methods*.

In the JSTDM version of HMSC, we included in the multivariate response matrix not only data on site‐level species abundances but also on their site‐level traits. Each species appears multiple times in the response data matrix, separate columns designated to species abundances and traits. Thus, the response matrix includes different response types (indexed by $z=1,\ldots ,n_{z}$; Figure 1) for each species, such as its abundance and its trait values for one or more traits. The extended linear predictor $L_{\mathrm{ijz}}$ then models the response types z of species j in site i through

(1) $$L_{\mathrm{ijz}}=\sum_{k}^{n_{c}}x_{\mathrm{ik}}\beta_{\mathrm{kjz}}+\sum_{k}^{n_{f}}\eta_{\mathrm{ik}}\lambda_{\mathrm{kjz}}.$$

Equation (1) assumes that the different response types may respond dissimilarly to both measured and latent predictors, as $\beta_{\mathrm{kjz}}$ (the responses to the $n_{c}$ measured predictors $x_{\mathrm{ik}}$) and $\lambda_{\mathrm{kjz}}$ (the responses to the $n_{f}$ latent factors $\eta_{\mathrm{ik}}$) are specific not only to the species j but also to the response type z. Our core interest is in the covariance matrix generated by the latent loadings $\lambda_{\mathrm{kjz}}$, the matrix Ω now containing associations among different response types ($z_{1}$ and $z_{2}$) of different species ($j_{1}$ and $j_{2}$):

(2) $$\Omega_{\left[j_{1},z_{1}\right],\left[j_{2},z_{2}\right]}=\sum_{k}^{n_{f}}\lambda_{kj_{1}z_{1}}\lambda_{kj_{2}z_{2}},$$

Assuming that the response type $z_{1}$ would be species abundance and the response type $z_{2}$ a particular trait, a positive (respectively, negative) estimate of $\Omega_{\left[j_{1},z_{1}\right],\left[j_{2},z_{2}\right]}$ would suggest that the species $j_{2}$ would have especially large (respectively, especially small) traits in sites where the species $j_{1}$ is abundant.

JSTDM models the expected response $\mu_{\mathrm{kjz}}=E\left[\beta_{\mathrm{kjz}}\right]$ of response type z of species j to environmental predictor k through species‐level traits,

(3) $$\mu_{\mathrm{kjz}}=\sum_{l}^{n_{t}}t_{\mathrm{jl}}\gamma_{\mathrm{klz}}.$$

Equation (3) assumes that the influence of species‐level traits on environmental responses may be different for different response types, as the responses $\gamma_{\mathrm{klz}}$ are specific not only to the environmental predictor k but also to its type z. The parameter $\gamma_{\mathrm{klz}}$ thus estimates how species‐level trait l influences the response of type z to the environmental predictor k. In case of the response type z being a trait, $\gamma_{\mathrm{klz}}$ models whether variation among species in environmentally generated variation in site‐level traits is related to species‐level traits, thus providing novel insights in relationships between species‐ and site‐level traits.

### Case study on tundra plants: A JSTDM model with 65 species and 5 response types

As a case study, we considered data on tundra plants studied on 325 sites, which in our case study are 1 m × 1 m plots. As detailed in Appendix S1: Section S2, we estimated abundance within each site as percentage coverage and measured the following site‐level traits: (1) specific leaf area, measured as the ratio of leaf area to leaf dry mass (henceforth SLA; unit in square centimeters per gram); (2) leaf area (henceforth LA; unit in square millimeters); and (3) median height, measured as the median vegetative height of the species within the site (henceforth MH; unit in centimeters). For each species within each site, we measured the three traits for three individuals (or for all individuals if less than three were present) and defined the site‐level traits as the average value. As environmental predictors, we compiled site‐level data on three microclimate variables: growing degree days (GDD), freezing degree days (FDD), and soil moisture (SM) (Appendix S1: Section S3).

Due to zero‐inflated nature of the data, we modeled abundances with a hurdle approach, the first part of the model predicting species occurrence, that is, whether the species was present at all or not, and the second part its abundance conditional on the species being present. These two components of the hurdle model jointly model variation in abundance since variation in species presence is a component of variation in species abundance. When referring to variation in abundance, we refer to both of these components. The main novelty of the method proposed here is that, in addition to abundances, the response matrix includes data on site‐level species traits (Figure 1). For the example case study, there are five response types ($n_{z}=5$) of each species: presence–absence (PA), abundance conditional on presence (ABUC), and the traits SLA, LA, and MH. We included in the analyses those 65 plant species that occurred in at least 25 out of the 325 sites (Appendix S1: Table S1). Hence, the response matrix had the dimensions of 325 outcomes (65 species × 5 response types) recorded in 325 sites.

HMSC enables the combination of different types of traits in a single multivariate model by allowing the specification of the error distribution and link function separately for each outcome (Ovaskainen & Abrego, 2020). We modeled presence–absence with a Bernoulli distribution and the probit link function and the other four outcomes (ABUC, SLA, LA, and MH) with a log‐normal distribution. Furthermore, HMSC allows for missing data in the response matrix, which is highly relevant for JSTDM as trait values will be missing for sites where the species is absent.

We fitted two JSTDM models to the data: A “null model,” including the intercept only, and an “environmental model,” including the environmental predictors (GDD, FDD, and SM). Both models included species abundances and site‐level traits as responses, with latent factors capturing associations among these ($\Omega_{\left[j_{1},z_{1}\right],\left[j_{2},z_{2}\right]}$, see Figure 1 and Equation 2). The environmental model further contained species‐level traits (trait values averaged across all sites) to model the influences of species‐level traits ($\gamma_{\mathrm{klz}}$, see Figure 1 and Equation 3) on how each response type was influenced by the environmental covariates ($\beta_{\mathrm{kjz}}$, see Figure 1 and Equation 1). In the null model, the matrix Ω captures the raw statistical associations among the occurrences, abundances, and trait values, whereas in the environmental model, it captures residual associations that remain after controlling for the effects of environmental predictors. The comparison between the Ω matrices derived from the null model and the environmental model thus allows to evaluate which trait‐abundance associations are, and which are not, explained by environmental variation. In particular, the residual associations in the environmental model may reflect patterns of trait‐driven intra‐ and inter‐specific interactions. However, this interpretation is non‐conclusive, as with non‐manipulative data any associations can also be generated by processes not included in the model, such as missing environmental predictors.

We fitted the models with the R‐package Hmsc‐R (Tikhonov et al., 2020) assuming the default prior distribution of the Bayesian model HMSC (for details, see chapter 8 in Ovaskainen & Abrego, 2020). We ran four Markov chain Monte Carlo (MCMC) chains for 37,500 iterations, of which we dropped the first 12,500 iterations as transient and thinned the remaining chains by 100 to yield 250 posterior samples per chain and 1000 posterior samples in total. To examine how much knowledge on species abundances helps in predicting their traits, we applied 10‐fold cross‐validation among the sites to the environmental model. We compared the predictive performances of a baseline cross‐validation, which does not utilize information about associations between species traits and their abundances, and a conditional cross‐validation, which utilizes such information when making predictions on species traits. In the baseline cross‐validation, we masked the entire response vector for the test fold, whereas for conditional cross‐validation, we masked data on species traits but not data on their abundances. As the measure of predictive performance, we used the correlation between predicted and observed values, calculated separately for each species‐trait combination. We note that the square of the correlation equals the *R* ^2^ of the underlying linear model, but we did not take the square as that would not separate whether the prediction is better (positive correlation) or worse (negative correlation) than by random.

To quantify how much species occurrences are influenced by environmental variation, the abundances of other species, and the trait values of other species, we considered scenarios that varied not only in environmental conditions but also in the abundance and trait values of *E. nigrum* subsp. *hermaphroditum* (henceforth *E. nigrum*). The reason why we selected *E. nigrum* as the focal species is that it has been previously reported to affect the performance and composition of the neighboring plant communities (Bråthen et al., 2010; le Roux et al., 2013; Mod et al., 2016; Pellissier et al., 2010). *E. nigrum* can have both negative effects via allelopathy and resource competition (Nilsson, 1994) and positive effects due to engineering warmer microclimatic conditions and stabilizing geomorphological processes affecting neighboring species (Carlsson & Callaghan, 1991). For each of the scenarios, we predicted the occurrences of all other species except *E. nigrum* and summarized the predictions in terms of species richness, obtained as the sum of species‐specific occurrence probabilities.

## RESULTS

### Associations among species abundances and traits

**FIGURE 2:**
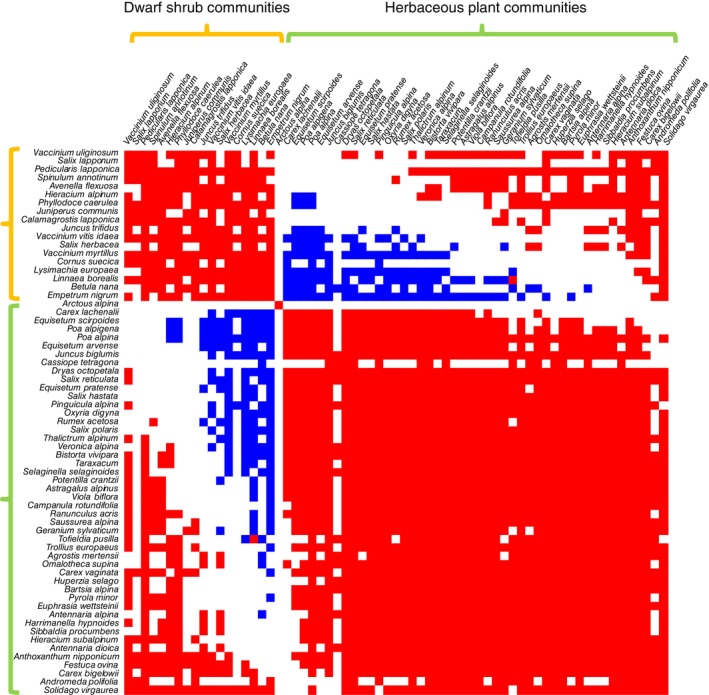
Residual associations among species presence–absences, as quantified by the matrix Ω of the fitted Hmsc model. The species have been ordered in a way that depicts the association structure most clearly, enabling the identification of two distinct community types that were negatively associated with each other. In the first community type, the proportion of woody plants was 58%, whereas in the second community type, it was only 17%. We refer to these as dwarf shrub communities and the herbaceous plant communities, respectively. The colors indicate cases for which the posterior probability of the association being positive (red) or negative (blue) is at least 90%. For a corresponding plot for raw associations (derived from the null model) rather than residual associations (derived from the environmental model), see Appendix S1: Figure S2.

**FIGURE 3:**
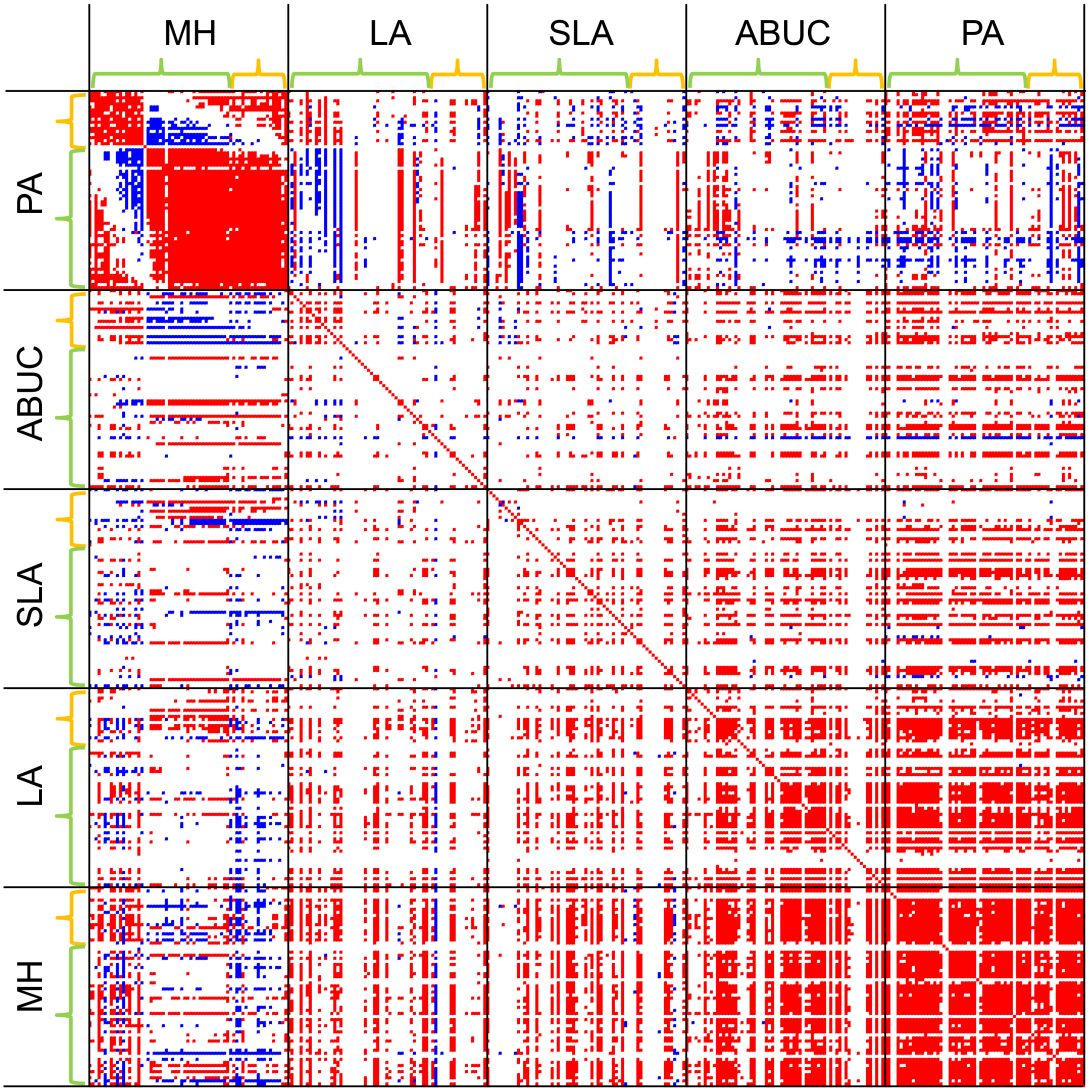
Residual associations among species abundances and traits, as quantified by the matrix Ω of the fitted Hmsc model. The figure shows associations among presence–absences (PA), abundance conditional on presence (ABUC), and three traits (LA, leaf area; MH, median vegetative height; SLA, specific leaf area). Within each of these five groups, the species have been ordered in the same order as in Figure 2. The colors indicate cases for which the posterior probability of the association being positive (red) or negative (blue) is at least 90%. The names and traits of the species are given in Appendix S1: Table S1. For a corresponding plot for raw associations (derived from the null model) rather than residual associations (derived from the environmental model), see Appendix S1: Figure S3.

The environmental model captured many statistically supported associations within and between species occurrences, abundances, and traits (Figures 2 and 3). As expected, the null model captured more associations than the environmental model (Appendix S1: Figures S2 and S3), showing that many of the associations detected by the null model were explained by responses of the species to the environmental conditions.

The associations among species occurrences identified two groups of species that showed positive associations within the groups but negative associations between the groups (Figure 2). Based on their species compositions, we call these groups the dwarf shrub communities and the herbaceous plant communities. The most common species in the dwarf shrub communities were *Vaccinium vitas‐idaea* (prevalence 67%) and *E. nigrum* (prevalence 59%), whereas the most common species in the herbaceous plant communities were *Bistorta vivipara* (prevalence 45%) and *Solidago virgaurea* (prevalence 43%). Some sites were thus characterized by a high number of species from the dwarf shrub community (henceforth, dwarf shrub sites), whereas some other sites were characterized by a high number of species from the herbaceous plant community (henceforth, herbaceous plant sites), even if these two types of communities showed also a degree of overlap. The environmental model further indicated that species belonging to the dwarf shrub communities were especially abundant in dwarf shrub sites, whereas they had especially low abundances in herbaceous plant sites in cases where they were present there (Figure 3). Associations among all three traits were predominantly positive among the species in both models (Figure 3). Thus, in some sites, the species had generally high values in all traits, whereas in some other sites, the species had generally low values in all traits. The associations between species presence–absences and their traits showed both positive and negative associations. In the environmental model, the clearest pattern was that dwarf shrub sites tended to have high MH traits for most species. The associations among ABUC and species traits were predominantly positive and organized in Figure 3 as red horizontal lines rather than vertical lines. This means that the abundances of some but not all species were positively associated with the trait values of all species.

For species occurrences and abundances, the most influential environmental predictors were GDD and FDD (Appendix S1: Figures S4 and S6). The occurrence probabilities of almost equally many species increased and decreased with respect to GDD, while for FDD, the occurrence probability of the majority of species increased (Appendix S1: Figure S4). For species traits, both GDD and FDD generally had a positive effect, the former mainly on MH and the latter on all three traits considered (Appendix S1: Figure S4). In both the null and the environmental models, the first, and thus most influential latent factor (Appendix S1: Figures S4, S5 and S6), modeled especially the positive associations among the traits (as indicated by factor loadings having the same sign for most traits) as well as either positive or negative associations between species presence–absences and traits. In both the null and the environmental models, the second and third latent factors modeled especially the species co‐occurrence patterns at the level of presence–absence.

### The interplay between species‐level traits and site‐level traits

While associations between species‐level traits and environmental responses are estimated already by the baseline HMSC model and have been reported in many earlier studies (Ovaskainen, Tikhonov, Norberg, et al., 2017), a novelty of the JSTDM extension of HMSC is that it enables also the estimation of how species‐level traits influence site‐level trait variation across environmental gradients ($\gamma_{\mathrm{klz}}$, see Figure 1 and Equation 3). In our case study, we detected a positive association among environmental covariate GDD, species‐level trait LA, and site‐level trait LA (Appendix S1: Figure S7). This indicates that those species whose site‐level LA values increased most with GDD had large species‐level LA values. Furthermore, we detected a positive association among environmental covariate GDD, species‐level trait LA, and site‐level presence–absence (Appendix S1: Figure S7). This indicates that species with high LA values were especially likely to be present in sites with high value of GDD. Jointly, these findings reveal an integrating picture: at sites with high GDD, there are especially many species with high species‐level LA values, and these species also tend to especially have large LA traits in those high GDD sites. This example demonstrates how the JSTDM provides insights not only by disentangling the interspecific and intraspecific components of trait variation but also by linking these two levels to each other.

### Knowledge on species abundance helps in predicting their traits

**FIGURE 4:**
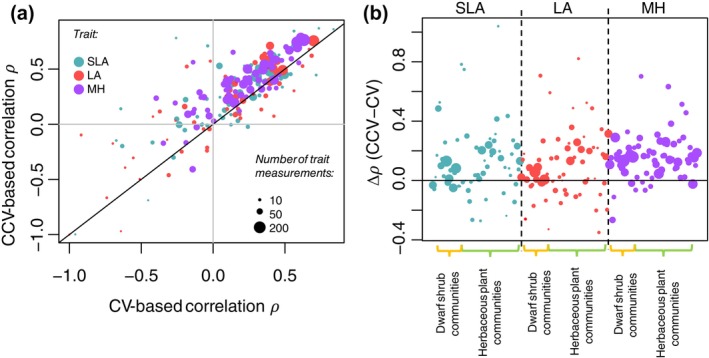
A comparison between predictive powers based on cross‐validation (CV) and conditional cross‐validation (CCV). As a measure of predictive power, we use the correlation between the predicted and actual values, with negative values indicating cases where the predictions are worse than by random. The three colors correspond to SLA = specific leaf area (dark cyan), LA = leaf area (red), and MH = median vegetative height (purple). Dot size is proportional to the amount of data, that is, number of trait measurements. In panel (a), the black line is identity line so that for cases above the line, information on species abundances helps in predicting their traits. The gray lines show zero correlation. Panel (b) shows the same data as the difference between the two types of cross validation.

A comparison between predictive powers based on cross‐validation and conditional cross‐validation showed that using information on species abundances improved the predictions for the majority of the species (69% of the species for SLA, 66% for LA, and 88% for MH), as compared to predictions based on environmental predictors only (Figure 4). There was, however, much variation among the species: counting only cases where the difference in the correlation was at least 0.2, knowledge on traits improved the predictions for 28% of the species for SLA, 28% of the species for LA, and 29% of the species for MH. Conversely, including information on species abundances made the predictions at least 0.2 units worse only for 5% of the species for SLA, 6% of the species for LA, and 2% for MH.

### Relative roles of environmental predictors and Empetrum traits on species composition

**FIGURE 5:**
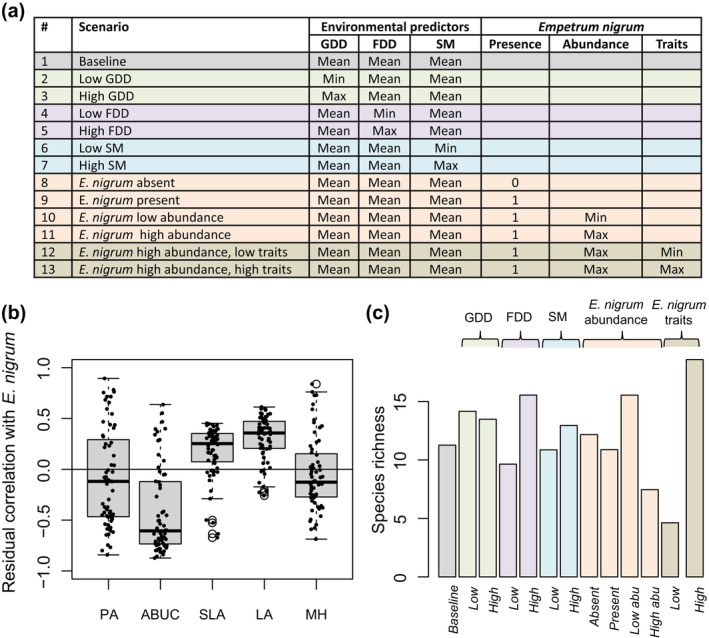
Associations between *Empetrum nigrum* and other species in the community. Panel (a) lists the scenarios used to disentangle the influences of environmental predictors and *E. nigrum* abundance and traits on the occurrences of the other species. The environmental predictors are abbreviated as GDD (growing degree days), FDD (freezing degree days), and SM (soil moisture). Empty cells indicate that the predictions are unconditional with respect to the variable. Panel (b) shows posterior mean estimates of residual association (covariances scaled to correlations) between the occurrences of all other species (each species shown by a dot), and the occurrence (PA), abundance conditional on presence (ABUC), and the traits SLA = specific leaf area, LA = leaf area, and MH = median vegetative height of *E. nigrum*. Panel (c) shows the posterior mean of predicted species richness under the scenarios.

The occurrence probabilities of most species were negatively associated with *E. nigrum* abundance (especially ABUC) and positively associated with *E. nigrum* SLA and LA traits, whereas the occurrences of almost equally many species were associated negatively and positively with *E. nigrum* MH (Figure 5). The predicted species richness under the scenarios (Figure 5c) reflects the estimated associations (Figure 5b): species richness was especially low if *E. nigrum* was abundantly present, was short, and had small leaves (Figure 5; scenario 12). Interestingly, the influence of trait values was so strong that the highest species richness among the scenarios was predicted in the case where *E. nigrum* was abundantly present, was tall, and had large leaves (Figure 5; scenario 13). Notably, variation in species richness among *E. nigrum*‐related scenarios (Figure 5; scenarios 8–13) was higher than variation in species richness among environmental condition‐related scenarios (Figure 5; scenarios 2–7).

## DISCUSSION

How to efficiently link intraspecific trait variation with species abundance variation in species‐rich communities has remained a large methodological challenge in community ecology. Several metrics have been developed to capture phenotypic variation across individuals within species, populations, or communities (Carmona, De Bello, et al., 2019; Cianciaruso et al., 2009; De Bello et al., 2011; Fontana et al., 2016; Valladares et al., 2006; Wong & Carmona, 2021). However, the metrics do not directly address how traits and abundances influence each other; each metric focuses on different aspects of intraspecific variation, and some of the metrics are sensitive to the number of sampling units and the number of species included in the analysis (Carmona, De Bello, et al., 2019; Wong & Carmona, 2021). Directly modeling trait values avoids some of these issues (Sandel et al., 2021), but such approaches have been earlier applied separately for each species‐trait combination, failing to account for covariances among traits and abundances for data where multiple traits are measured for multiple species. Alternatively, species abundances may be predicted using site‐level trait information (Laughlin et al., 2012), but this approach uses traits as predictors for abundances, rather than explicitly addressing their co‐variation. Here, we have developed an efficient methodological workflow within the JSDM framework to explicitly link species abundances to phenotypic variation in traits for multiple species simultaneously.

As we demonstrate with a case study of tundra plants, the methodology presented in this study allows assessing the interplay between species abundances and traits at the community level. In particular, the estimated association matrix among abundances and traits (matrix Ω, Figure 2) allows assessing which traits increase and decrease in relation to the abundance of a given species or vice versa. These relationships may reflect direct or indirect interactive relationships among individuals or joint responses among abundances and traits to unmeasured environmental variables. In our case study, the association matrices revealed two types of species communities, some sites being dominated by dwarf shrubs and other sites by herbaceous plants. Regardless of the differences in species composition, the association plots showed consistencies across trait values so that in some sites the species had generally high values of all traits, whereas in some other sites, the species had generally low values of all traits. While it is not possible to make conclusive inference on processes based on observational data, a likely explanation of these patterns is that dwarf shrub dominant species may outcompete herbaceous plant communities, and that synchronous trait variation among all species may represent differences in the soil nutrient availability across the sites.

A major advantage of modeling traits directly as the response variable, rather than first summarizing them into indices, is that it allows making predictions, which can be used for multiple purposes. In our case study, we made two types of predictions. On the one hand, we predicted plant trait values conditional on data on species abundances, the results showing that information on species abundances generally improves predictions for traits. Since collecting site‐level trait data for multiple species across multiple sites is highly resource‐intensive, our results suggest that a viable option is to collect abundance data for all sites but trait data only for a subset of sites and then impute the trait data for the remaining sites using conditional prediction. On the other hand, we used scenario simulations to assess to what extent the traits of the focal *E. nigrum* species influenced the abundances and richness of its neighboring species. The results showed that the occurrence probabilities of most species were associated negatively with *E. nigrum* abundances but positively with *E. nigrum* SLA and LA traits. The first pattern, which is consistent with multiple SDM‐based studies where the abundance of *E. nigrum* has been included as a covariate, could indicate the competitive and allopathic influences of *E. nigrum* on other species (le Roux et al., 2013; Mod et al., 2016; Pellissier et al., 2010). The latter result may reflect two alternative, though not mutually exclusive, mechanisms. It might be that under conditions where *E. nigrum* has high trait values, the nutrient levels are also high, which may be beneficial to plants in general, leading to higher species richness. Testing for this hypothesis would require also adding site‐level soil nutrient data to the model. Another plausible explanation is that when *E. nigrum* is taller, it also tends to grow less dense (i.e., *E. nigrum's* aboveground biomass does not increase linearly with the height), leaving more space in between the branches and letting more light through its canopy for other species.

Methodological literature on JSDM has extensively discussed the fact that, with non‐manipulative data on species abundances, it is not possible to conclusively infer whether the estimated associations reflect species interactions or confounding effects of missing environmental predictors (Blanchet et al., 2020; Dormann et al., 2018; Ovaskainen & Abrego, 2020; Ovaskainen et al., 2019; Zurell et al., 2018). If applied to non‐manipulative data, the same fundamental limitation of not being possible to infer processes from data patterns also holds to JSTDM. However, co‐variation between fitness‐related traits and species abundances can be expected to be more informative about species interactions than merely co‐variation in species abundances. While in this study we used snapshot data as a case study to illustrate the methodology, the same statistical methods can equally be applied to time‐series data (Ovaskainen, Tikhonov, Dunson, et al., 2017), which may give more dynamic information about the relationships between traits and abundances. Yet we stress that to obtain conclusive evidence on the underlying processes, experimental approaches are needed, for instance, trait manipulation experiments with selective removal or addition of individuals (Bennett et al., 2016; Holden & Cahill, 2024). The methodology presented here could be applied also to experimental data, to gain a more predictive understanding of how traits and abundances influence the traits and abundance of focal individuals.

Previous experimental research on plant communities has linked species distribution modeling with trait variation by the ΔTraitSDMs method, where data on reciprocal transplantations are used to disentangle trait variation into components related to local adaptation and phenotypic plasticity, and then this information is used to predict areas outside the species current range where the species could potentially be able to persist in the future (Benito Garzón et al., 2019). While the species distribution modeling part of the ΔTraitSDMs method is based on making assumptions about the role of species traits in the viability and abundance of the local populations, the methodology proposed here directly identifies such links. Thus, these two methods are highly complementary, and using them in combination provides exciting avenues for future research, such as making more reliable predictions of expected species range shifts in response to climate change.

The methodology developed in this study responds to the growing demand of incorporating phenotypic trait variation to unveil the mechanisms of community assembly. The methodology developed here also offers exciting avenues to analyze other types of multiresponse multivariate data, such as analyses of metatranscriptomic data combined with species abundance data. One could, for example, link trait variation to community functional diversity or ecosystem functioning by assessing how gene expressions associate with species occurrences or abundances under varying environmental conditions.

## CONFLICT OF INTEREST STATEMENT

The authors declare no conflicts of interest.

## Supporting information


Appendix S1:

## Data Availability

Data and code (Abrego, 2025) are available on Zenodo at https://doi.org/10.5281/zenodo.15280766.

## References

[ecy70174-bib-0001] Abrego, N. 2025. “Data and Scripts for ‘Joint Species‐Trait Distribution Modelling: The Role of Intraspecific Trait Variation in Community Assembly’ [Data Set].” Zenodo. 10.5281/zenodo.15280766.40908572

[ecy70174-bib-0002] Abrego, N. , A. Norberg , and O. Ovaskainen . 2017. “Measuring and Predicting the Influence of Traits on the Assembly Processes of Wood‐Inhabiting Fungi.” Journal of Ecology 105(4): 1070–1081. 10.1111/1365-2745.12722.

[ecy70174-bib-0003] Benito Garzón, M. , T. M. Robson , and A. Hampe . 2019. “ΔTrait SDMS: Species Distribution Models that Account for Local Adaptation and Phenotypic Plasticity.” New Phytologist 222(4): 1757–1765. 10.1111/nph.15716. 30697749

[ecy70174-bib-0004] Bennett, J. A. , K. Riibak , R. Tamme , R. J. Lewis , and M. Pärtel . 2016. “The Reciprocal Relationship between Competition and Intraspecific Trait Variation.” Journal of Ecology 104(5): 1410–1420. 10.1111/1365-2745.12614.

[ecy70174-bib-0005] Blanchet, F. G. , K. Cazelles , and D. Gravel . 2020. “Co‐Occurrence Is Not Evidence of Ecological Interactions.” Ecology Letters 23(7): 1050–1063. 10.1111/ele.13525. 32429003

[ecy70174-bib-0006] Bolnick, D. I. , P. Amarasekare , M. S. Araújo , R. Bürger , J. M. Levine , M. Novak , V. H. W. Rudolf , S. J. Schreiber , M. C. Urban , and D. A. Vasseur . 2011. “Why Intraspecific Trait Variation Matters in Community Ecology.” Trends in Ecology & Evolution 26(4): 183–192. 10.1016/j.tree.2011.01.009. 21367482 PMC3088364

[ecy70174-bib-0007] Bråthen, K. A. , C. H. Fodstad , and C. Gallet . 2010. “Ecosystem Disturbance Reduces the Allelopathic Effects of *Empetrum hermaphroditum* Humus on Tundra Plants: Ecosystem Disturbance Reduces Allelopathic Effects.” Journal of Vegetation Science 21: 786–795. 10.1111/j.1654-1103.2010.01188.x.

[ecy70174-bib-0008] Brown, A. M. , D. I. Warton , N. R. Andrew , M. Binns , G. Cassis , and H. Gibb . 2014. “The Fourth‐Corner Solution – Using Predictive Models to Understand How Species Traits Interact with the Environment.” Methods in Ecology and Evolution 5(4): 344–352. 10.1111/2041-210X.12163.

[ecy70174-bib-0009] Carlsson, B. A. , and T. V. Callaghan . 1991. “Positive Plant Interactions in Tundra Vegetation and the Importance of Shelter.” The Journal of Ecology 79(4): 973. 10.2307/2261092.

[ecy70174-bib-0010] Carmona, C. P. , F. Bello , F. M. Azcárate , N. W. H. Mason , and B. Peco . 2019. “Trait Hierarchies and Intraspecific Variability Drive Competitive Interactions in Mediterranean Annual Plants.” Journal of Ecology 107(5): 2078–2089. 10.1111/1365-2745.13248.

[ecy70174-bib-0011] Carmona, C. P. , F. De Bello , N. W. H. Mason , and J. Lepš . 2019. “Trait Probability Density (TPD): Measuring Functional Diversity across Scales Based on TPD with R.” Ecology 100(12): e02876. 10.1002/ecy.2876. 31471976

[ecy70174-bib-0012] Cianciaruso, M. V. , M. A. Batalha , K. J. Gaston , and O. L. Petchey . 2009. “Including Intraspecific Variability in Functional Diversity.” Ecology 90(1): 81–89. 10.1890/07-1864.1. 19294915

[ecy70174-bib-0013] Clark, J. S. 2016. “Why Species Tell More about Traits than Traits about Species: Predictive Analysis.” Ecology 97(8): 1979–1993. 10.1002/ecy.1453. 27859208

[ecy70174-bib-0014] De Bello, F. , S. Lavorel , C. H. Albert , W. Thuiller , K. Grigulis , J. Dolezal , Š. Janeček , and J. Lepš . 2011. “Quantifying the Relevance of Intraspecific Trait Variability for Functional Diversity.” Methods in Ecology and Evolution 2(2): 163–174. 10.1111/j.2041-210X.2010.00071.x.

[ecy70174-bib-0015] Dormann, C. F. , M. Bobrowski , D. M. Dehling , D. J. Harris , F. Hartig , H. Lischke , M. D. Moretti , et al. 2018. “Biotic Interactions in Species Distribution Modelling: 10 Questions to Guide Interpretation and Avoid False Conclusions.” Global Ecology and Biogeography 27(9): 1004–1016. 10.1111/geb.12759.

[ecy70174-bib-0016] Doudová, J. , and J. Douda . 2020. “Along with Intraspecific Functional Trait Variation, Individual Performance Is Key to Resolving Community Assembly Processes.” Functional Ecology 34(11): 2362–2374. 10.1111/1365-2435.13646.

[ecy70174-bib-0017] Fontana, S. , O. L. Petchey , and F. Pomati . 2016. “Individual‐Level Trait Diversity Concepts and Indices to Comprehensively Describe Community Change in Multidimensional Trait Space.” Functional Ecology 30(5): 808–818. 10.1111/1365-2435.12551.

[ecy70174-bib-0018] Freschet, G. T. , E. M. Swart , and J. H. C. Cornelissen . 2015. “Integrated Plant Phenotypic Responses to Contrasting Above‐ and Below‐Ground Resources: Key Roles of Specific Leaf Area and Root Mass Fraction.” New Phytologist 206(4): 1247–1260. 10.1111/nph.13352. 25783781

[ecy70174-bib-0019] Gonçalves‐Souza, T. , L. S. Chaves , G. X. Boldorini , N. Ferreira , R. A. F. Gusmão , P. B. Perônico , N. J. Sanders , and F. B. Teresa . 2023. “Bringing Light onto the Raunkiæran Shortfall: A Comprehensive Review of Traits Used in Functional Animal Ecology.” Ecology and Evolution 13(4): e10016. 10.1002/ece3.10016. 37091571 PMC10115901

[ecy70174-bib-0020] Holden, E. M. , and J. F. Cahill . 2024. “Plant Trait Dissimilarity Increases Competitive Interactions among Co‐Occurring Plants.” Functional Ecology 38: 1365‐2435.14561. 10.1111/1365-2435.14561.

[ecy70174-bib-0021] Kemppinen, J. , and P. Niittynen . 2022. “Microclimate Relationships of Intraspecific Trait Variation in Sub‐Arctic Plants.” Oikos 2022(12): e09507. 10.1111/oik.09507.

[ecy70174-bib-0022] Körner, C. 2007. “The Use of “Altitude” in Ecological Research.” Trends in Ecology & Evolution 22(11): 569–574. 10.1016/j.tree.2007.09.006. 17988759

[ecy70174-bib-0023] Kraft, N. J. B. , G. M. Crutsinger , E. J. Forrestel , and N. C. Emery . 2014. “Functional Trait Differences and the Outcome of Community Assembly: An Experimental Test with Vernal Pool Annual Plants.” Oikos 123(11): 1391–1399. 10.1111/oik.01311.

[ecy70174-bib-0024] Kuppler, J. , C. H. Albert , G. M. Ames , W. S. Armbruster , G. Boenisch , F. C. Boucher , D. R. Campbell , et al. 2020. “Global Gradients in Intraspecific Variation in Vegetative and Floral Traits Are Partially Associated with Climate and Species Richness.” Global Ecology and Biogeography 29(6): 992–1007. 10.1111/geb.13077.

[ecy70174-bib-0025] Laughlin, D. C. , C. Joshi , P. M. Van Bodegom , Z. A. Bastow , and P. Z. Fulé . 2012. “A Predictive Model of Community Assembly that Incorporates Intraspecific Trait Variation.” Ecology Letters 15(11): 1291–1299. 10.1111/j.1461-0248.2012.01852.x. 22906233

[ecy70174-bib-0026] le Roux, P. C. , J. Lenoir , L. Pellissier , M. S. Wisz , and M. Luoto . 2013. “Horizontal, but Not Vertical, Biotic Interactions Affect Fine‐Scale Plant Distribution Patterns in a Low‐Energy System.” Ecology 94(3): 671–682. 10.1890/12-1482.1. 23687893

[ecy70174-bib-0027] Mod, H. K. , R. K. Heikkinen , P. C. Le Roux , M. S. Wisz , and M. Luoto . 2016. “Impact of Biotic Interactions on Biodiversity Varies across a Landscape.” Journal of Biogeography 43(12): 2412–2423. 10.1111/jbi.12794.

[ecy70174-bib-0028] Nilsson, M.‐C. 1994. “Separation of Allelopathy and Resource Competition by the Boreal Dwarf Shrub *Empetrum hermaphroditum* Hagerup.” Oecologia 98(1): 1–7. 10.1007/BF00326083. 28312789

[ecy70174-bib-0029] Ovaskainen, O. , and N. Abrego . 2020. Joint Species Distribution Modelling: With Applications in R, 1st ed. Cambridge and New York: Cambridge University Press. 10.1017/9781108591720.

[ecy70174-bib-0030] Ovaskainen, O. , J. Rybicki , and N. Abrego . 2019. “What Can Observational Data Reveal about Metacommunity Processes?” Ecography 42(11): 1877–1886. 10.1111/ecog.04444.

[ecy70174-bib-0031] Ovaskainen, O. , G. Tikhonov , D. Dunson , V. Grøtan , S. Engen , B.‐E. Sæther , and N. Abrego . 2017. “How Are Species Interactions Structured in Species‐Rich Communities? A New Method for Analysing Time‐Series Data.” Proceedings of the Royal Society B: Biological Sciences 284(1855): 20170768. 10.1098/rspb.2017.0768. PMC5454278 28539525

[ecy70174-bib-0032] Ovaskainen, O. , G. Tikhonov , A. Norberg , F. G. Blanchet , L. Duan , D. Dunson , T. Roslin , and N. Abrego . 2017. “How to Make More Out of Community Data? A Conceptual Framework and Its Implementation as Models and Software.” Ecology Letters 20(5): 561–576. 10.1111/ele.12757. 28317296

[ecy70174-bib-0033] Pellissier, L. , K. A. Bråthen , J. Pottier , C. F. Randin , P. Vittoz , A. Dubuis , N. G. Yoccoz , T. Alm , N. E. Zimmermann , and A. Guisan . 2010. “Species Distribution Models Reveal Apparent Competitive and Facilitative Effects of a Dominant Species on the Distribution of Tundra Plants.” Ecography 33(6): 1004–1014. 10.1111/j.1600-0587.2010.06386.x.

[ecy70174-bib-0034] Pollock, L. J. , W. K. Morris , and P. A. Vesk . 2012. “The Role of Functional Traits in Species Distributions Revealed through a Hierarchical Model.” Ecography 35(8): 716–725. 10.1111/j.1600-0587.2011.07085.x.

[ecy70174-bib-0035] Poorter, H. , Ü. Niinemets , L. Poorter , I. J. Wright , and R. Villar . 2009. “Causes and Consequences of Variation in Leaf Mass per Area (LMA): A Meta‐Analysis.” New Phytologist 182(3): 565–588. 10.1111/j.1469-8137.2009.02830.x. 19434804

[ecy70174-bib-0036] Rahbek, C. 1995. “The Elevational Gradient of Species Richness: A Uniform Pattern?” Ecography 18(2): 200–205. 10.1111/j.1600-0587.1995.tb00341.x.

[ecy70174-bib-0037] Rixen, C. , S. Wipf , S. B. Rumpf , J. Giejsztowt , J. Millen , J. W. Morgan , A. B. Nicotra , et al. 2022. “Intraspecific Trait Variation in Alpine Plants Relates to Their Elevational Distribution.” Journal of Ecology 110(4): 860–875. 10.1111/1365-2745.13848.

[ecy70174-bib-0038] Sandel, B. , C. Pavelka , T. Hayashi , L. Charles , J. Funk , F. W. Halliday , G. S. Kandlikar , et al. 2021. “Predicting Intraspecific Trait Variation among California's Grasses.” Journal of Ecology 109(7): 2662–2677. 10.1111/1365-2745.13673.

[ecy70174-bib-0039] Siefert, A. , C. Violle , L. Chalmandrier , C. H. Albert , A. Taudiere , A. Fajardo , L. W. Aarssen , et al. 2015. “A Global Meta‐Analysis of the Relative Extent of Intraspecific Trait Variation in Plant Communities.” Ecology Letters 18(12): 1406–1419. 10.1111/ele.12508. 26415616

[ecy70174-bib-0040] Sundqvist, M. K. , N. J. Sanders , and D. A. Wardle . 2013. “Community and Ecosystem Responses to Elevational Gradients: Processes, Mechanisms, and Insights for Global Change.” Annual Review of Ecology, Evolution, and Systematics 44(1): 261–280. 10.1146/annurev-ecolsys-110512-135750.

[ecy70174-bib-0041] Tikhonov, G. , Ø. H. Opedal , N. Abrego , A. Lehikoinen , M. M. J. Jonge , J. Oksanen , and O. Ovaskainen . 2020. “Joint Species Distribution Modelling with the R‐package HMSC.” Methods in Ecology and Evolution 11(3): 442–447. 10.1111/2041-210X.13345. 32194928 PMC7074067

[ecy70174-bib-0042] Valladares, F. , D. Sanchez‐Gomez , and M. A. Zavala . 2006. “Quantitative Estimation of Phenotypic Plasticity: Bridging the Gap between the Evolutionary Concept and Its Ecological Applications.” Journal of Ecology 94(6): 1103–1116. 10.1111/j.1365-2745.2006.01176.x.

[ecy70174-bib-0043] Violle, C. , B. J. Enquist , B. J. McGill , L. Jiang , C. H. Albert , C. Hulshof , V. Jung , and J. Messier . 2012. “The Return of the Variance: Intraspecific Variability in Community Ecology.” Trends in Ecology & Evolution 27(4): 244–252. 10.1016/j.tree.2011.11.014. 22244797

[ecy70174-bib-0044] Warton, D. I. , F. G. Blanchet , R. B. O'Hara , O. Ovaskainen , S. Taskinen , S. C. Walker , and F. K. C. Hui . 2015. “So Many Variables: Joint Modeling in Community Ecology.” Trends in Ecology & Evolution 30(12): 766–779. 10.1016/j.tree.2015.09.007. 26519235

[ecy70174-bib-0045] Wong, M. K. L. , and C. P. Carmona . 2021. “Including Intraspecific Trait Variability to Avoid Distortion of Functional Diversity and Ecological Inference: Lessons from Natural Assemblages.” Methods in Ecology and Evolution 12(5): 946–957. 10.1111/2041-210X.13568.

[ecy70174-bib-0046] Wright, I. J. , P. B. Reich , M. Westoby , D. D. Ackerly , Z. Baruch , F. Bongers , J. Cavender‐Bares , et al. 2004. “The Worldwide Leaf Economics Spectrum.” Nature 428(6985): 821–827. 10.1038/nature02403. 15103368

[ecy70174-bib-0047] Zurell, D. , L. J. Pollock , and W. Thuiller . 2018. “Do Joint Species Distribution Models Reliably Detect Interspecific Interactions from Co‐Occurrence Data in Homogenous Environments?” Ecography 41(11): 1812–1819. 10.1111/ecog.03315.

