# Supplementary material for: Joint species‐trait distribution modeling: The role of intraspecific trait variation in community assembly
Source: Ecology. 2025 Sep 4;106(9):e70174. doi: 10.1002/ecy.70174 (PMC12411695; doi:10.1002/ecy.70174)
Supplement: Supplementary file 1 — Appendix S1: [file ECY-106-e70174-s001.pdf]

Appendix S1

Joint species-trait distribution modeling: The role of intraspecific trait variation in community assembly

Nerea Abrego, Pekka Niittynen, Julia Kemppinen and Otso Ovaskainen

Ecology

Table of contents

Section S1: The baseline HMSC model 2
Section S2: Data on tundra plant communities 3
Figure S1 4
Section S3: Data on environmental conditions 5
Section S4: Supporting results 6
Table S1 6
Figure S2 9
Figure S3 10
Figure S4 11
Figure S5 12
Figure S6 13
Figure S7 14
References 15

Section S1: The baseline HMSC model

The baseline HMSC models species occurrences or abundances through the linear predictor L_{ij}, where j is the index of the species (with $j = 1, \ldots, n_s$), and i is the index for the plot (with $i = 1, \ldots, n_y$). Through different link functions and error distributions, HMSC can incorporate data on presence-absences (probit regression), counts (log-normal Poisson distribution), or continuous responses (normal distribution). The linear predictor is modeled as

$$L_{ij} = \sum_{k}^{n_c} x_{ik}\beta_{kj} + \sum_{k}^{n_f} \eta_{ik}\lambda_{kj}, \qquad \text{(Eq. S1)}$$

where x_{ik} are measured predictors, η_{ik} are latent predictors, and β_{kj} and λ_{kj} are regression coefficients quantifying responses of the species to the measured and the latent predictors. The latent predictors induce within-plot dependence across species abiotic or biotic conditions not captured by the x_{ik}s (Ovaskainen et al. 2016). From the fitted models, our main interest was in the variance-covariance matrix (the matrix Ω)(Ovaskainen et al. 2017; Ovaskainen and Abrego 2020) generated by the latent loadings, as this matrix quantifies how variation in intraspecific trait values relates to abundance variation. Following from the usual assumption $\eta_{ik} \sim N(0,1)$ of factor analysis, the latent loadings of the species generate the covariance(Ovaskainen et al. 2017; Ovaskainen and Abrego 2020)

$$\Omega_{j_1 j_2} = \sum_{k}^{n_f} \lambda_{kj_1}\lambda_{kj_2}, \qquad \text{(Eq. S2)}$$

between species j_1 and j_2 at the level of the linear predictor. The species-to-species association matrix Ω then describes which species are positively or negatively associated to each other, beyond their responses to the environmental predictors x_{ik}.

HMSC enables for the identification of trait-environment relationships by modeling the expected response $\mu_{kj} = E[\beta_{kj}]$ of species j to environmental predictor k through species level traits,

$$\mu_{kj} = \sum_{l}^{n_t} t_{jl}\gamma_{kl} \qquad \text{(Eq. S3)}$$

Here, t_{jl} is the trait l of species j, and γ_{kl} is the influence of trait l on the expected response of a species on the predictor k. HMSC models variation of species responses β_{kj} by a multivariate normal distribution, the mean of which is given by the μ_{kj}, and the variance-covariance matrix of which may be structured by phylogenetic relationships among the species.

Section S2: Data on tundra plant communities

Vascular plant data were collected in the Kilpisjärvi region, spanning approximately 150 square kilometers, known as a sub-Arctic biodiversity hotspot situated in northwestern Finland (Kauhanen 2013). The area is predominantly characterized by sub-Arctic tundra, where mountain birch (*Betula pubescens* ssp. *chzerepanovii*) forests thrive at lower altitudes. Dwarf shrubs such as *Empetrum nigrum, Betula nana*, and various species of *Vaccinium* constitute the most prevalent and abundant species. Graminoids and forbs, though diverse, primarily flourish in moist meadows or other specific and spatially more restricted habitats.

The plant data encompass plant abundance and trait records obtained from 325 one square-meter (1m x 1m) plots (Fig. S1). The plots comprehensively capture the primary environmental gradients of the area, such as elevation (ranging from 473 to 1029 meters above sea level), slope aspect, soil moisture and snow conditions. Within each plot, we identified all vascular plant species present and visually estimated their percentage coverage as indicative of their abundance. As traits, we measured the median vegetative heights (MH), leaf area (LA) and specific leaf area (SLA) of the different species within the plots (Fig S1). For each species within each plot, we measured the three traits for three individuals (or for all individuals if less than three were present) and defined the plot-level traits as the average value.

MH measures the plant's ability to compete for light (Westoby 1998). Sub-Arctic tundra plants are generally low-statured (Bjorkman et al. 2018), yet the plant communities can form several relatively dense canopy layers, resulting in decreased light interception towards the ground surface. Larger leaves, instead, allow plants to compete for light by expanding horizontally. SLA varies along the well-documented leaf economics spectrum, which characterizes multivariate correlations of leaf functional traits along a single axis of variation (Wright et al. 2004). SLA typically correlates with nutrient contents of leaves, and is associated for example with plants' photosynthetic efficiency and growth rates (Shipley et al. 2005; Poorter and Bongers 2006). Arctic-alpine non-woody species typically exhibit relatively high SLA as they need to grow rapidly to complete their life cycles within the short growing season (Choler 2005). Large and thin leaves (high SLA) maximize the photosynthetic surface area per unit of dry matter, but they also result in higher evaporative surface and increased susceptibility to environmental stressors such as drought or freezing. High SLA is associated with a strategy where plants invest many resources into rapid growth rather than conserving resources to tolerate stress (Choler 2005; Onipchenko et al. 2023).

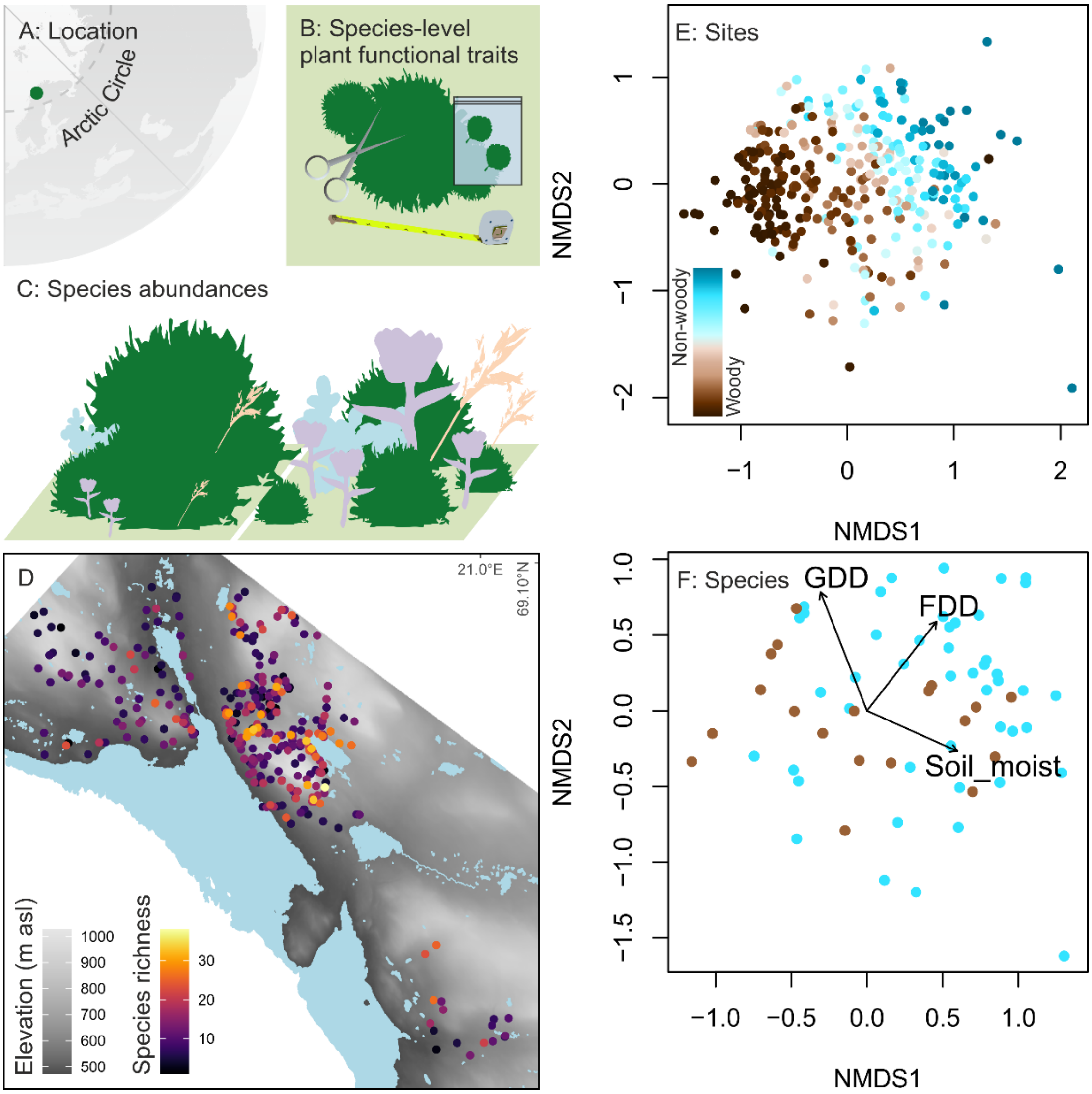

Figure S1. Case study on arctic tundra plants. The study area is located in the Arctic (A). Species traits (B) and abundances (C) were measured at 1 m^2 sample plots along an elevational gradient (D). In panel (E), the color depicts the proportion of woody species in each plot, showing that the sample plots cluster in the NMDS ordination space into woody and non-woody communities. In panel (F), the woody (brown) and non-woody (cuan) species are shown in the NMDS space with the environmental predictors of growing degree days (GDD), freezing degree days (FDD) and soil moisture (Soil_moist).

Section S3: Data on environmental conditions

We considered three microclimate variables to explain the distributions and traits of the focal tundra plant communities: Growing degree days (GDD) of near-surface air temperature (hereafter growing degree days), mean summer soil moisture (hereafter soil moisture) and freezing degree days of near-surface air temperature (hereafter freezing degree days) (Fig. S1). Growing degree days represents the cumulative sum of daily mean temperatures when the mean temperature exceeds 3 degrees Celsius, indicating the overall warmth and duration of the growing season. Soil moisture denotes the volumetric water content (%) of the topsoil during the growing season, reflecting local water availability for plants. Freezing degree days (FDD) represents the cumulative sum of daily mean temperatures when the mean temperature falls below zero, summarizing the severity of winter conditions. These microclimate variables, or similar ones, have demonstrated significance in elucidating patterns in species occurrences or trait variation among plant species inhabiting cold environments (Choler 2005; P. Niittynen, Heikkinen, and Luoto 2020; Opedal, Armbruster, and Graae 2015; Kemppinen and Niittynen 2022). These environmental predictors stem from a fine-scale gridded microclimate dataset published by some of the authors (Pekka Niittynen et al. 2024). Detailed information regarding the raw microclimate data, methodologies, and derived variables can be found in Niittynen et al. (2024) and briefly below.

MH (excluding floral parts) was measured using a ruler. For LA and SLA, we collected leaf samples from three individual ramets from all species covering more than one percent in the plot. If fewer than three plants were present in the plot or immediate vicinity, we collected leaves from all available individuals. The leaves were stored in a moist and cool environment until processing, typically for less than 24 hours. We scanned the fresh leaves using a photo scanner, then dried them in an oven at 70°C for 48 hours before weighing them with a high-precision scale (Mettler AE 100; 0.0001 g precision). LA was measured using ImageJ software and the LeafArea R package based on the scanned images (Katabuchi 2015). Finally, we calculated SLA, defined as the ratio of dry weight to leaf area.

In summary, the microclimate dataset comprises data from over 400 microclimate loggers, measuring year-round soil temperatures (-6cm) and near-surface air temperatures (+15cm), and soil moisture when the soil is not frozen. The logger data were used to calculate 73 microclimate variables (e.g., monthly mean temperatures), which were related to a suite of gridded environmental variables (e.g., elevation, topographic wetness index, remotely sensed snow cover duration), and subsequently predicted across the entire study area. In the study area, growing degree days exhibits a strong negative correlation with elevation ($R_{Pearson}$ = -0.87). Freezing degree days, although negatively correlated with elevation (-0.47), displays a weaker association due to its closer relationship with local snow conditions. Soil moisture shows minimal association with elevation (0.04).

Section S4: Supporting results

Table S1. List of the 65 plant species included in the case study, ordered as in Figs. 2-3 of the main document. The column C (community type) describes whether in our classification (based of the ordering of species in Figs. 2-3 of the main document) the species belongs to dwarf shrub communities (S) or herbaceous plant communities (H). The column W (woodiness) describes whether the species is woody (1) or non-woody (0). The abundance of the plants is described by their prevalence across the plots (%) and mean (me), minimun (mi) and maximun (ma) abundance across those plots where they are present (ABUC). The traits are characterized by the mean (me), minimun (mi) and maximun (ma) values of the specific leaf area (SLA), leaf area (LA) and mean height (MH) across those plots where the species are present.

	C	W	%	ABUC me	ABUC mi	ABUC ma	SLA me	SLA mi	SLA ma	LA me	LA mi	LA ma	MH me	MH mi	MH ma
Vaccinium uliginosum	S	1	37	5.71	0.25	35	12.4	9.5	22.8	1	0.3	2.3	6.9	1	19
Salix lapponum	S	1	13	3.11	0.25	35	12.5	10.3	15.4	4.8	1.2	7.5	18.5	4	85
Pedicularis lapponica	S	0	17	0.36	0.25	1	18.8	12	22.9	2	1.1	3.1	3.2	1	12
Spinulum annotinum	S	0	8	1.41	0.25	7	13.6	8.3	17.7	0	0	0.1	4.7	1	9
Avenella flexuosa	S	0	41	3.15	0.25	40	11	5	19.4	0.6	0.3	1.1	6.9	2	18
Hieracium alpinum	S	0	11	0.86	0.25	9	21.1	15.8	24.6	2.9	1.9	3.8	3.2	1	11
Phyllodoce caerulea	S	1	30	4.59	0.25	30	9.1	7.2	11.8	0.1	0.1	0.6	5.2	1	11
Juniperus communis	S	1	13	8.22	0.25	55	5.5	4	8.4	0.1	0	0.1	27.3	4	92
Calamagrostis lapponica	S	0	21	1.08	0.25	8	18.9	14.5	24.1	3.6	1.9	6.1	13.6	6	25
Juncus trifidus	S	0	19	1.01	0.25	5	10.7	4.1	16.4	0.3	0.1	0.5	8	2	17
Vaccinium vitis idaea	S	1	67	3.27	0.25	25	6.9	4.5	15	0.7	0.2	2	3.9	0.7	12
Salix herbacea	S	1	27	3.95	0.25	23	12.4	9	19.8	1.2	0.4	3.5	1.7	0.5	5
Vaccinium myrtillus	S	1	40	8.57	0.25	55	17.3	12.8	26.8	0.9	0.3	1.9	8.2	2	17
Cornus suecica	S	0	9	5.14	0.5	40	25.1	19.4	31.8	2.3	1.2	4.6	6.8	3	12
Lysimachia europaea	S	0	18	0.9	0.25	4	32.8	28	42.5	1.4	0.4	2.9	2.7	0.5	7
Betula nana	S	1	40	7.48	0.25	45	12.4	8.6	24.9	1	0.3	2	13.5	0.5	61
Linnaea borealis	S	1	22	1.75	0.25	15	17.9	13.2	27.9	0.4	0.2	0.6	1.4	0.5	4
Empetrum nigrum	S	1	59	13.18	0.25	75	7	4.8	11.6	0.1	0	0.4	5.4	1	13
Arctous alpina	S	1	8	3.75	0.25	20	12.3	10.5	15.3	1.1	0.8	1.7	2.7	1	5
Carex lachenalii	H	0	9	1.04	0.25	5	19.1	15.4	23.6	1.1	0.6	2.5	5.9	2	13

	C	W	%	ABUC me	ABUC mi	ABUC ma	SLA me	SLA mi	SLA ma	LA me	LA mi	LA ma	MH me	MH mi	MH ma
Equisetum scirpoides	H	0	10	1.16	0.25	20	9.2	8.3	10.5	0.5	0.3	0.7	4.4	1	11
Poa alpigena	H	0	12	1.47	0.25	20	19.8	14	29.2	3.7	1.3	8.3	12.2	3	25
Poa alpina	H	0	18	1.1	0.25	7	15.5	10.2	20.8	2.8	0.8	6.9	5.2	2	15
Equisetum arvense	H	0	8	1.82	0.25	20	17.4	11.5	26.2	0.3	0.1	0.6	7.3	2.5	21
Juncus biglumis	H	0	9	0.4	0.25	1	15.6	14.8	16.4	0.2	0.2	0.3	3.9	2	8
Cassiope tetragona	H	1	17	3.77	0.25	30	6.7	4.3	10.2	0.1	0	0.1	5.6	2	13
Dryas octopetala	H	1	10	19.05	0.5	85	11.1	7.9	16.4	0.9	0.3	2.2	2.5	1	5
Salix reticulata	H	1	11	4.99	0.25	25	10.7	8.5	16.2	3.6	0.9	10.5	2.6	0.5	4
Equisetum pratense	H	0	10	1.37	0.25	6	18.1	14.7	25.9	0.2	0.1	0.3	9.2	3	17
Salix hastata	H	1	13	1.7	0.25	15	14.2	9.7	20.1	3.9	1.7	6.2	8.5	1	50
Pinguicula alpina	H	0	10	0.47	0.25	1	43.9	43.1	44.7	1.3	1.2	1.4	0.6	0.3	1
Oxyria digyna	H	0	9	3.31	0.25	17	21.7	16.3	39.8	2.7	1.1	4.9	2.6	1	6
Rumex acetosa	H	0	16	1.27	0.25	5	33.2	21.6	60.4	7.6	2.2	17.8	5.1	2	15
Salix polaris	H	1	14	4.29	0.25	30	14.4	10.5	26	1.4	0.5	3.8	1.7	0.5	5
Thalictrum alpinum	H	0	22	3.68	0.25	30	17.6	13	26.2	2.5	0.9	4.7	3.6	1	8
Veronica alpina	H	0	17	0.4	0.25	2	20.5	16.5	24.2	0.5	0.2	0.8	3.6	0.5	15
Bistorta vivipara	H	0	45	2.84	0.25	15	17.7	12.3	35.1	3.5	1	8.8	4	1	10
Taraxacum	H	0	24	1.8	0.25	15	31.1	21.6	48.2	5.5	2.6	12.1	3.3	0.5	11
Selaginella selaginoides	H	0	22	0.45	0.25	3	32	23.6	37.7	0	0	0	2.8	0.5	7
Potentilla crantzii	H	0	10	1.39	0.25	8	16.5	9.6	22.2	3.2	1.6	6.6	3.6	1	16
Astragalus alpinus	H	0	13	2.8	0.25	11	28.6	18	55.8	4	0.2	9.6	3.6	1	9
Viola biflora	H	0	39	5.24	0.25	55	42.3	31.8	69.4	4.3	1.3	13.9	2.3	0.5	12
Campanula rotundifolia	H	0	14	0.86	0.25	10	23.3	15.8	31.1	1.4	0.5	2.2	4.1	1	15
Ranunculus acris	H	0	12	1.22	0.25	7	27.7	19.7	38.5	3.1	1.4	5.2	3.8	1	15
Saussurea alpina	H	0	31	3.74	0.25	20	20.2	11.1	35.2	11.6	1.9	26.9	5.9	1	15
Geranium sylvaticum	H	0	16	2.64	0.25	30	25.6	15.6	41.1	18.4	1.8	50.1	7.6	0.5	30
Tofieldia pusilla	H	0	8	0.55	0.25	2	13.1	10.6	15.7	0.3	0.2	0.3	1.7	1	3
Trollius europaeus	H	0	19	3.76	0.25	25	21.6	15.3	32.5	18.4	2.9	52	6.4	1	19

	C	W	%	ABUC me	ABUC mi	ABUC ma	SLA me	SLA mi	SLA ma	LA me	LA mi	LA ma	MH me	MH mi	MH ma
Agrostis mertensii	H	0	10	0.5	0.25	2	22.4	16.6	26.9	0.7	0.5	0.9	5.6	1	15
Omalotheca supina	H	0	16	0.8	0.25	10	30.8	21.6	42.6	0.2	0.1	0.4	1.1	0.4	4
Carex vaginata	H	0	35	2.2	0.25	25	19.1	14.4	25	3.8	1.2	10.3	7.2	1	18
Huperzia selago	H	0	9	0.31	0.25	0.5	NA	NA	NA	NA	NA	NA	2.2	0.5	7
Bartsia alpina	H	0	14	1.13	0.25	6	18.2	14.8	21.9	1.1	0.6	1.9	8.2	3	17
Pyrola minor	H	0	10	0.95	0.25	4	17.7	15.8	20.4	3.4	1.7	4.9	1.9	0.5	4
Euphrasia wettsteinii	H	0	14	0.38	0.25	2	33.5	27.2	41.5	0.2	0.2	0.3	3.4	0.5	10
Harrimanella hypnoides	H	1	12	5.53	0.25	30	13.3	4.1	21.6	0	0	0	0.7	0.2	2
Antennaria alpina	H	0	10	0.73	0.25	4	18.1	13.9	23	0.3	0.2	0.4	1.2	0.5	3
Sibbaldia procumbens	H	1	10	1.19	0.25	6	18.5	14.7	26.8	1.7	0.9	3.6	1.9	0.5	4
Hieracium subalpinum	H	0	14	0.66	0.25	2	23.7	17.4	42	5.4	1.3	10.5	3.7	1	12
Antennaria dioica	H	0	10	3.17	0.25	35	16	13.3	21.5	0.5	0.2	1	1.7	1	3
Anthoxanthum nipponicum	H	0	26	1.32	0.25	10	23.8	17.3	35	3.2	1	5.5	9.8	2	22
Festuca ovina	H	0	40	1.18	0.25	15	9.8	6.5	13.9	0.4	0.1	1.4	4.9	0.5	11
Carex bigelowii	H	0	35	2.76	0.25	25	16.7	1.7	23.5	2.2	0.9	6.1	7.1	0.5	17
Andromeda polifolia	H	1	9	2.2	0.25	8	7.1	5.3	9.3	0.3	0.2	0.5	5	3	7
Solidago virgaurea	H	0	43	1.87	0.25	15	26.9	17.3	50.2	7.4	1.7	28	6.1	1	21

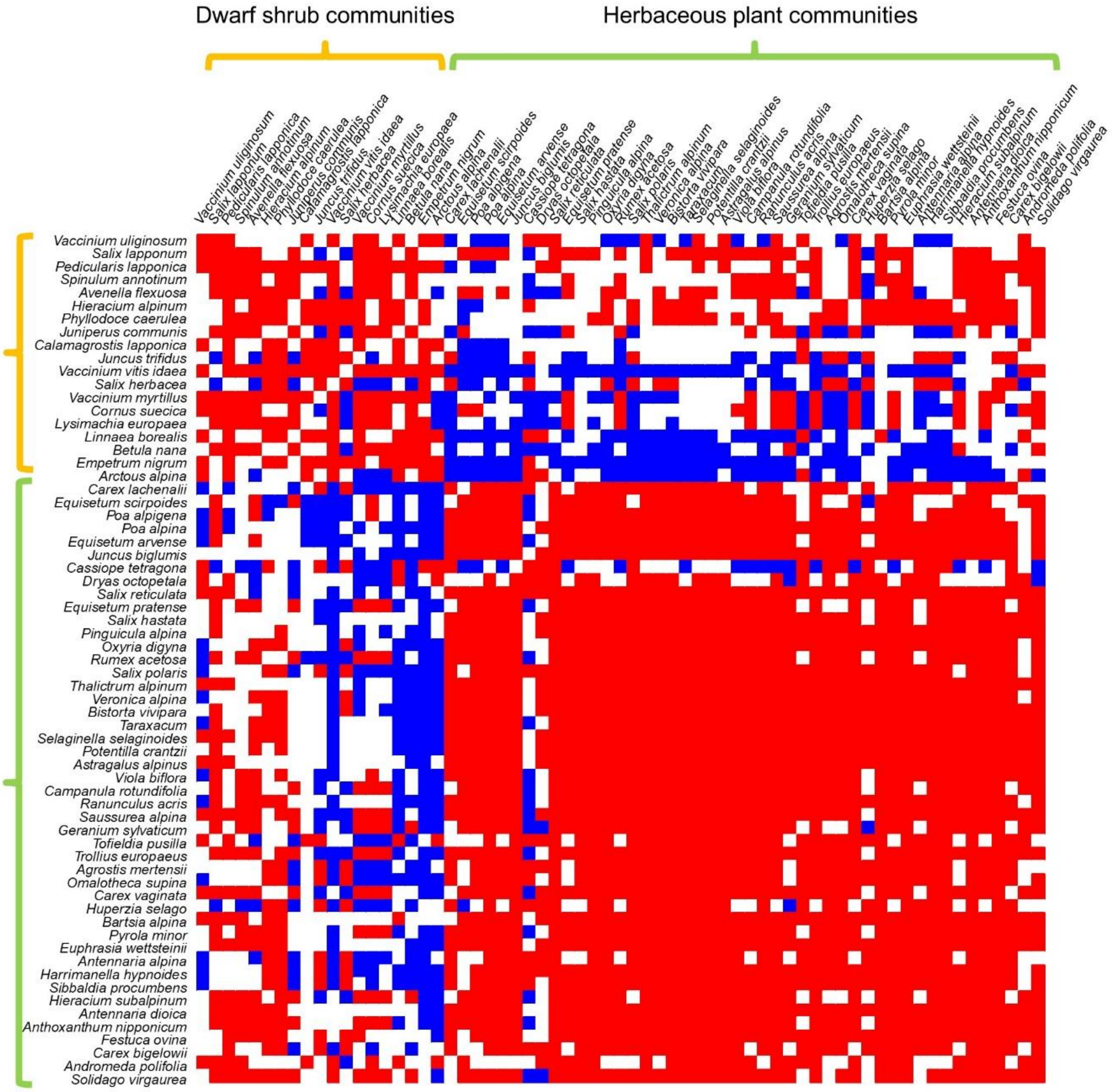

Figure S2. Raw associations among species presence-absences, as quantified by the matrix Ω of the fitted Hmsc-model. The species have been ordered in the same order as in Fig. 2 of the main text. The colors indicate cases for which the posterior probability of the association being positive (red) or negative (blue) is at least 90%.

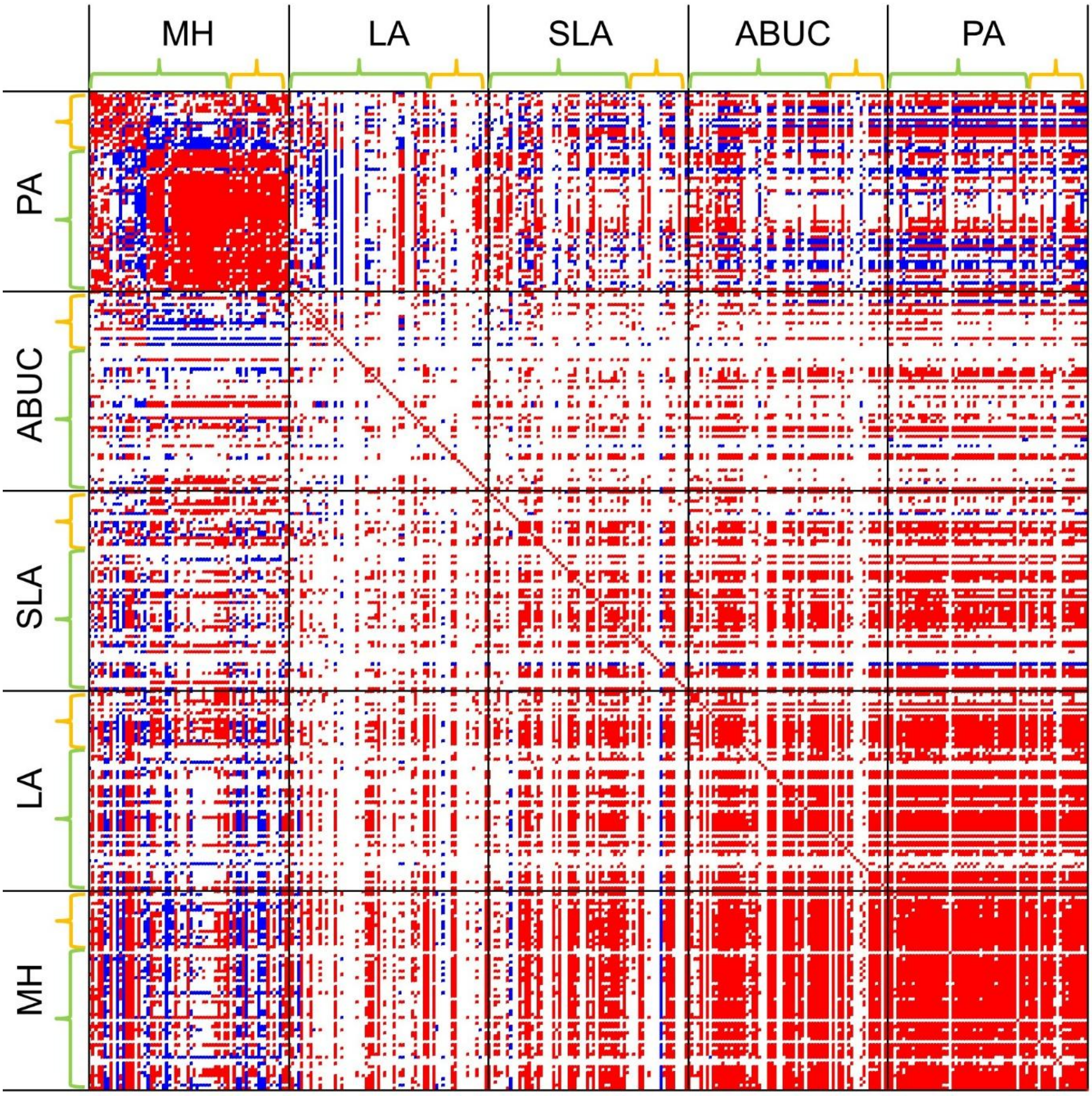

Figure S3. Raw associations among species abundances and traits, as quantified by the matrix Ω of the fitted Hmsc-model. The figure shows associations among presence-absences (PA), abundances conditional on presence (ABUC), and three traits (SLA = specific leaf area, LA = leaf area and MH = median vegetative height). Within each of these five groups, the species have been ordered in the same order as in Fig. 2 of the main text. The colors indicate cases for which the posterior probability of the association being positive (red) or negative (blue) is at least 90%. The names and traits of the species are given in Table S1. For a corresponding plot for residual associations (derived from the environmental model) rather than raw associations (derived from the null model), see Fig. 3 of the main text.

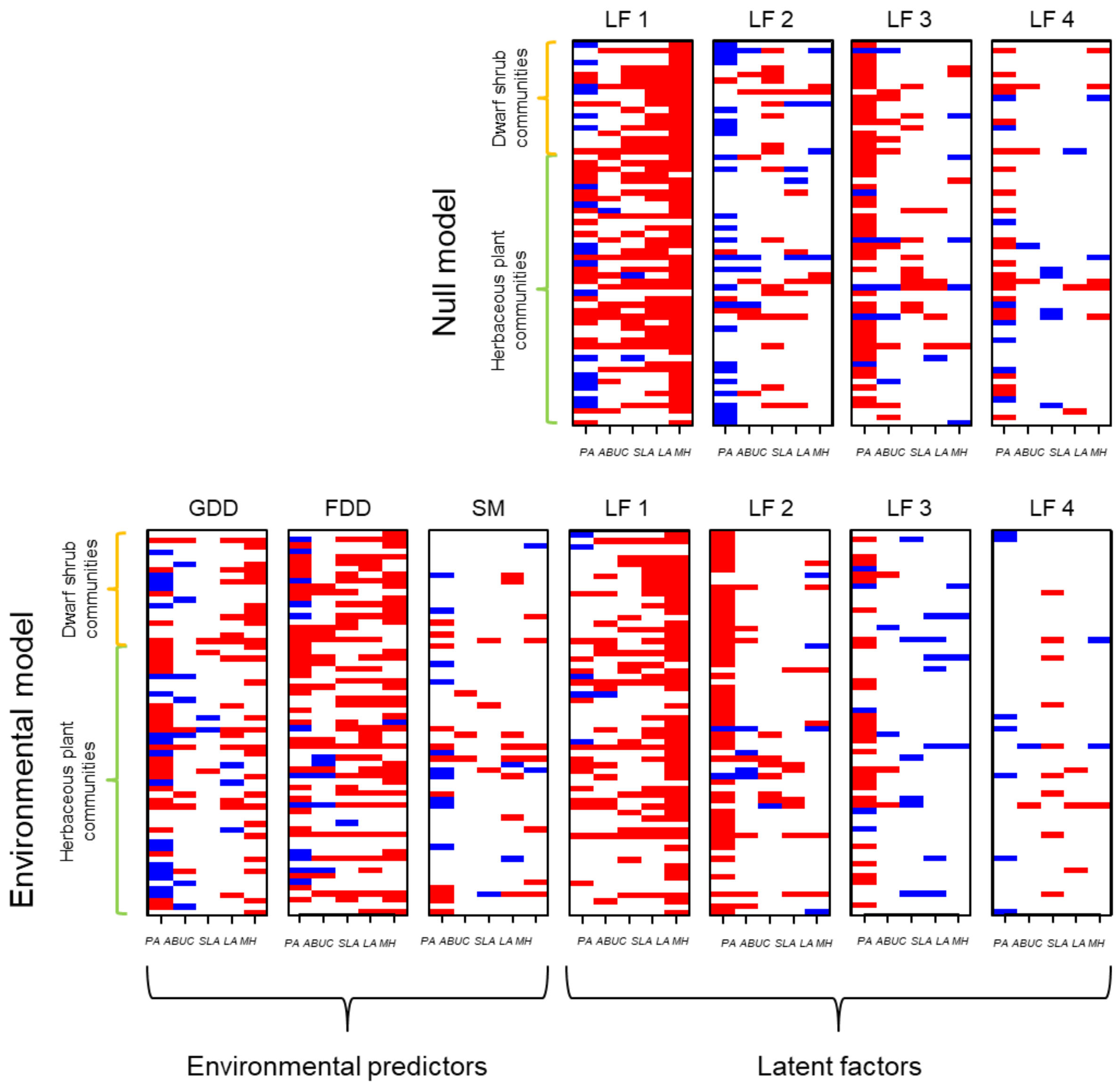

Figure S4. Responses of the species presence-absences, abundances conditional on presence, and traits on environmental and latent predictors. Responses that are positive with at least 95% posterior probability are shown by red, and responses that are negative with at least 95% posterior probability are shown by blue. Note that response sign for latent factor is arbitrary as multiplying both the latent factor and its loading vector by minus sign leads to an identical model. The names of the species are given in Table S1.

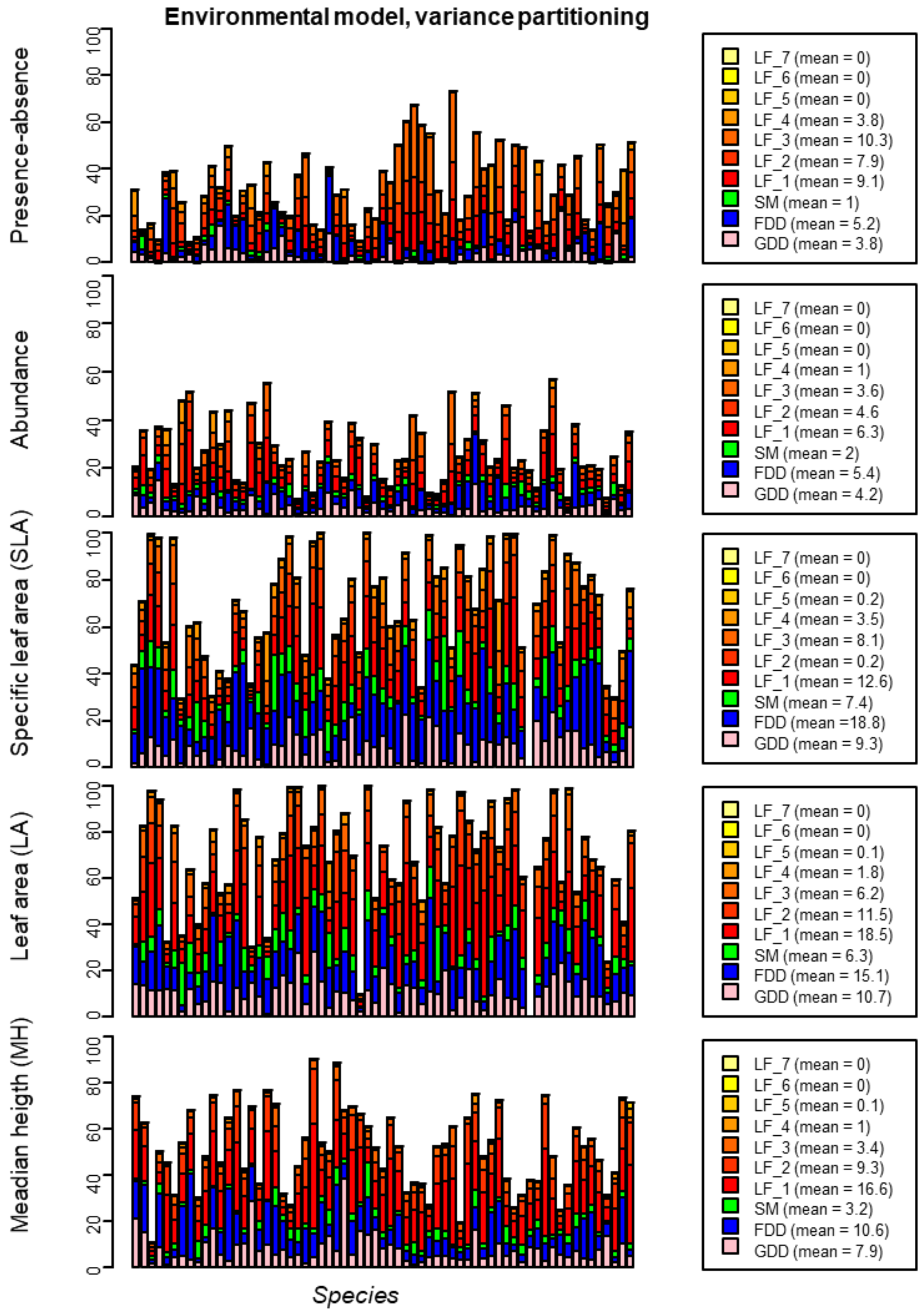

Figure S5. Variance partitioning among environmental and latent predictors in explaining species presence-absences, abundances conditional on presence, and traits in the environmental model. The names of the species are given in Table S1.

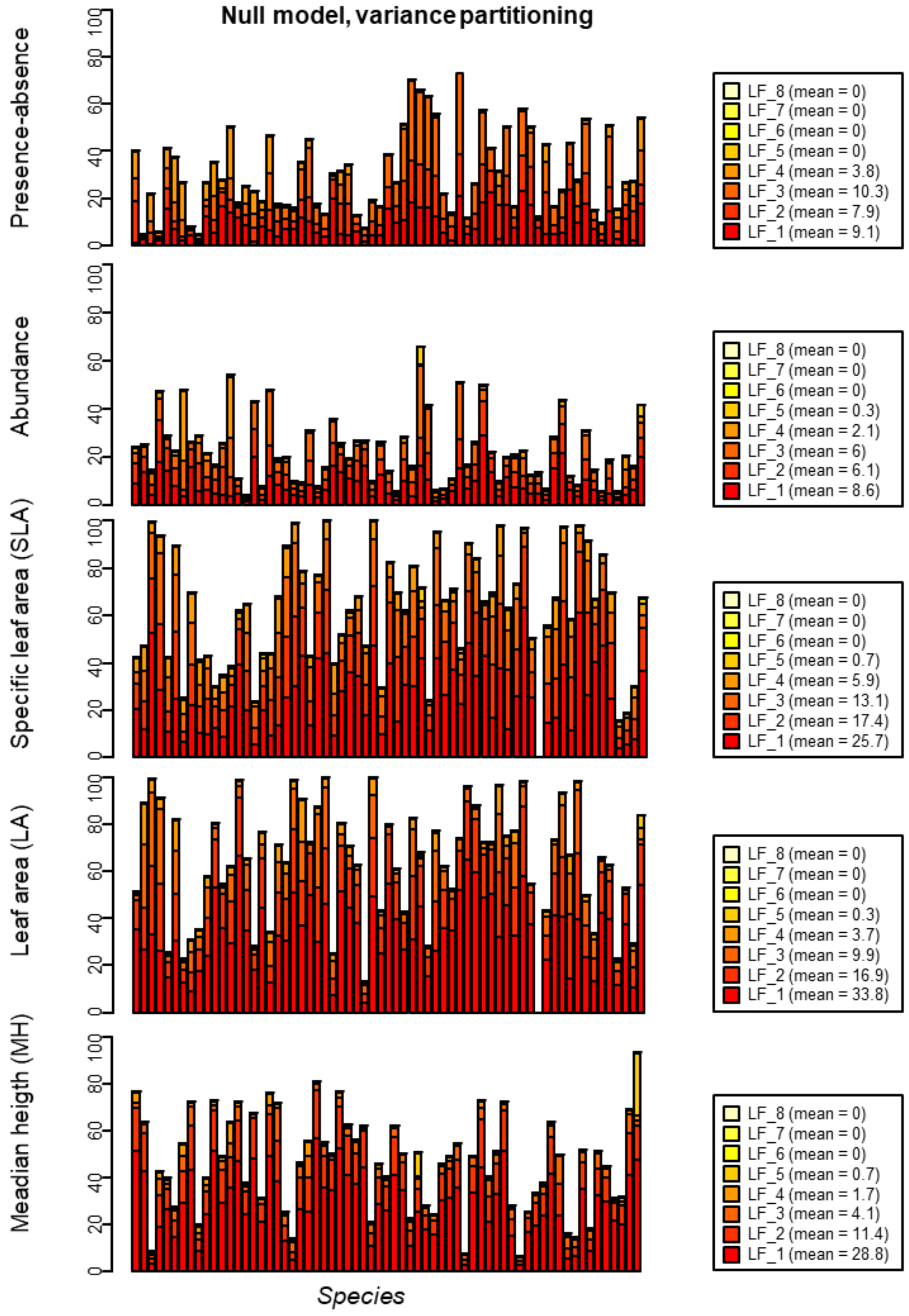

Figure S6. Variance partitioning among latent predictors in explaining species presence-absences, abundances conditional on presence, and traits in the null model. The names of the species are given in Table S1.

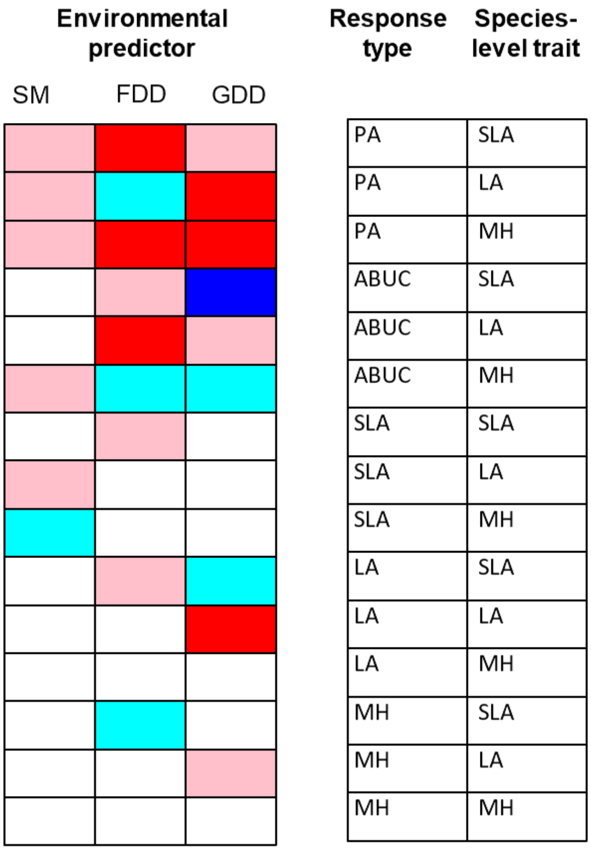

Figure S7. Estimated association parameters (γ_{klz}) between species-level traits, and the responses of species abundances and plot-level traits to environmental variation. Negative association are shown by dark blue (strong association; Pr<0.05) and light blue (weak association; Pr<0.25) and positive associations by red (strong association; Pr>0.95) and pink (weak association; Pr>0.75), where Pr is the posterior probability by which the estimate of γ_{klz} is positive.

References

Bjorkman, A. D., I. H. Myers-Smith, S. C. Elmendorf, S. Normand, N. Ruger, P. S. A. Beck, A. Blach-Overgaard, et al. 2018. ‘Plant Functional Trait Change across a Warming Tundra Biome’. *Nature* 562 (7725): 57–62. https://doi.org/10.1038/s41586-018-0563-7.

Choler, P. 2005. ‘Consistent Shifts in Alpine Plant Traits along a Mesotopographical Gradient’. *Arctic Antarctic and Alpine Research* 37 (4): 444–53. https://doi.org/10.1657/1523-0430(2005)037[0444:csiapt]2.0.co;2.

Katabuchi, Masatoshi. 2015. ‘LeafArea: An R Package for Rapid Digital Image Analysis of Leaf Area’. *Ecological Research* 30 (6): 1073–77. https://doi.org/10.1007/s11284-015-1307-x.

Kauhanen, Heikki O. 2013. ‘Mountains of Kilpisjärvi Host an Abundance of Threatened Plants in Finnish Lapland’. *J Botanica Pacifica: A Journal of Plant Science* 2 (1): 43–52.

Kemppinen, Julia, and Pekka Niittynen. 2022. ‘Microclimate Relationships of Intraspecific Trait Variation in sub-Arctic Plants’. *Oikos* 2022 (12): e09507. https://doi.org/10.1111/oik.09507.

Niittynen, P., R. K. Heikkinen, and M. Luoto. 2020. ‘Decreasing Snow Cover Alters Functional Composition and Diversity of Arctic Tundra’. *Proceedings of the National Academy of Sciences of the United States of America* 117 (35): 21480–87. https://doi.org/10.1073/pnas.2001254117.

Niittynen, P., H. Salminen, P. Peña-Aguilera, J. Aalto, J. Alahuhta, M. Luoto, T. Maliniemi, et al. 2024. ‘A Gridded Microclimate Dataset from a Sub-Arctic Biodiversity Hotspot in Finland’. bioRxiv. https://doi.org/10.1101/2024.03.30.587419.

Niittynen, Pekka, Henriikka Salminen, Pablo Peña-Aguilera, Juha Aalto, Janne Alahuhta, Miska Luoto, Tuija Maliniemi, et al. 2024. ‘A Dataset: Gridded Microclimate Dataset from a Sub-Arctic Biodiversity Hotspot in Finland’. https://doi.org/10.5281/zenodo.10897219.

Onipchenko, V. G., K. V. Dudova, D. M. Gulov, A. A. Akhmetzhanova, D. K. Tekeev, and T. G. Elumeeva. 2023. ‘Leaf Functional Traits Are Important for the Formation of Alpine Plant Community Composition’. *Biology Bulletin Reviews* 13 (3): 228–37. https://doi.org/10.1134/S2079086423030064.

Opedal, O. H., W. S. Armbruster, and B. J. Graae. 2015. ‘Linking Small-Scale Topography with Microclimate, Plant Species Diversity and Intra-Specific Trait Variation in an Alpine Landscape’. *Plant Ecology & Diversity* 8 (3): 305–15. https://doi.org/10.1080/17550874.2014.987330.

Ovaskainen, Otso, and Nerea Abrego. 2020. *Joint Species Distribution Modelling: With Applications in R*. 1st ed. Cambridge University Press. https://doi.org/10.1017/9781108591720.

Ovaskainen, Otso, Nerea Abrego, Panu Halme, and David Dunson. 2016. ‘Using Latent Variable Models to Identify Large Networks of Species-to-species Associations at Different Spatial Scales’. Edited by David Warton. *Methods in Ecology and Evolution* 7 (5): 549–55. https://doi.org/10.1111/2041-210X.12501.

Ovaskainen, Otso, Gleb Tikhonov, Anna Norberg, F. Guillaume Blanchet, Leo Duan, David Dunson, Tomas Roslin, and Nerea Abrego. 2017. ‘How to Make More out of Community Data? A Conceptual Framework and Its Implementation as Models and Software’. Edited by Jerome Chave. *Ecology Letters* 20 (5): 561–76. https://doi.org/10.1111/ele.12757.

Poorter, Lourens, and Frans Bongers. 2006. ‘Leaf Traits Are Good Predictors of Plant Performance across 53 Rain Forest Species’. *Ecology* 87 (7): 1733–43. https://doi.org/10.1890/0012-9658(2006)87[1733:LTAGPO]2.0.CO;2.

Shipley, B., D. Vile, E. Garnier, I. J. Wright, and H. Poorter. 2005. ‘Functional Linkages between Leaf Traits and Net Photosynthetic Rate: Reconciling Empirical and Mechanistic Models’. *Functional Ecology* 19 (4): 602–15. https://doi.org/10.1111/j.1365-2435.2005.01008.x.

Westoby, M. 1998. ‘A Leaf-Height-Seed (LHS) Plant Ecology Strategy Scheme’. *Plant and Soil* 199 (2): 213–27. https://doi.org/10.1023/a:1004327224729.

Wright, Ian J., Peter B. Reich, Mark Westoby, David D. Ackerly, Zdravko Baruch, Frans Bongers, Jeannine Cavender-Bares, et al. 2004. ‘The Worldwide Leaf Economics Spectrum’. *Nature* 428 (6985): 821–27. https://doi.org/10.1038/nature02403.
